# Supplementary material for: Evolution of channel flow and Darcy’s law beyond the critical Reynolds number
Source: Eur Phys J E Soft Matter. 2023 May 30;46(6):37. doi: 10.1140/epje/s10189-023-00289-4 (PMC10229703; doi:10.1140/epje/s10189-023-00289-4)
Supplement: Supplementary file 1 — Supplementary file1 (DOCX 21931 KB) [file 10189_2023_289_MOESM1_ESM.docx]

**Supplementary material for the article:**

**Evolution of channel flow and Darcy’s law beyond the critical Reynolds number**

Xiaohui Deng and Ping Sheng

I. Derivation of the 3D eigenmodes of incompressible Navier-Stokes equation in the channel geometry

The incompressible Navier-Stokes equation is given by

$\begin{matrix} \frac{\partial u\left(\mathbf{r}\right)}{\partial t}=-\nabla p\left(\mathbf{r}\right)+\frac{1}{\mathrm{Re}}\nabla^{2}u\left(\mathbf{r}\right), \mathbf{r}=\left(x,y,z\right) \\ \nabla\cdot u\left(\mathbf{r}\right)=0 \end{matrix}$ . (I.1)

Here $u$ denotes velocity, and$p$is the pressure. The channel geometry is defined by the solid boundaries at *z*=$\pm1$, with *z*=0 being the symmetry plane for categorizing the solutions as symmetric or antisymmetric. Navier slip boundary condition is imposed on the upper and lower solid/liquid interfaces; periodic boundary conditions in the *xy* plane are imposed on $u$ and$p$.

Generalized eigenfunctions of 3D incompressible fluid should have the form shown in (I.2) below. Here $m$ and $k$ are real, continuous variables, and both should be non-zero. For the same reason, $\tilde{u}_{x}\left( z \right)\neq0$  and  $\tilde{u}_{y}(z)\neq0$. As mentioned in the main text, in the absence of a natural length scale in the *xy* plane, such as that defined by the critical state wavevector value at Re_c_, or a length scale defined by the numerical computational domain, the periodicity in the *xy* plane is infinite.

(I.2)

In component form, the incompressiblity constraints may be written as:

(I.3a)

(I.3b)

(I.3c)

(I.3d)

The Navier boundary conditions are given by:

(I.4)

(I.5)

We show in the following that there are multiple linear dependency restrictions on the 12 coefficients in (I.2). That means the 12 coefficient cannot be uniquely solved.

In order to obtain the eigenfunctions of (I.1), which are laternatively denoted the hydrodynamic modes (HMs), we would like to first eliminate the pressure field by considering the vorticity equation

$$(\nabla^{2}+\lambda\mathrm{Re})\mathbf{\Omega}(\mathbf{r},t)=0 , \mathbf{\Omega}=\nabla\times u , (I.7)$$

where we have assumed that $u\propto e^{-\lambda t}$ and $\mathbf{\Omega}$ has the following components

It is noted that the pressure term is eliminated by considering the vorticity equation. However, the pressure can be recovered by solving the pressure Laplace equation$\nabla^{2}p=0$, with the boundary condition $\left\{\partial p/\partial z=(\mathrm{Re})^{-1}\partial^{2}u_{z}/\partial z^{2}\right\}_{z=\pm1}$, where the *u* denotes the HM. The pressure solution is given for each HM below.

In order to better observe the linear relation between the components$\tilde{u}_{x}(z)$, $\tilde{u}_{y}(z)$ and $\frac{d\tilde{u}_{z}(z)}{dz}$, we transform (I.3a-d) to the following form:

$$\begin{matrix} \left(A_{1}B_{3}+A_{2}B_{4}\right)m\tilde{u}_{x}(z)=-\left(B_{3}C_{3}+B_{4}C_{4}\right)\frac{d\tilde{u}_{z}(z)}{dz} (I.11a) \\ \left(A_{1}B_{3}+A_{2}B_{4}\right)k\tilde{u}_{y}(z)=-\left(A_{1}C_{4}-A_{2}C_{3}\right)\frac{d\tilde{u}_{z}(z)}{dz} (I.11b) \\ \left(A_{3}B_{1}+A_{4}B_{2}\right)m\tilde{u}_{x}(z)=\left(B_{1}C_{1}+B_{2}C_{2}\right)\frac{d\tilde{u}_{z}(z)}{dz} \begin{matrix} \\ \end{matrix} (I.11c) \\ \left(A_{3}B_{1}+A_{4}B_{2}\right)k\tilde{u}_{y}(z)=-\left(A_{3}C_{2}-A_{4}C_{1}\right)\frac{d\tilde{u}_{z}(z)}{dz} (I.11d) \end{matrix}$$

Before proceeding to simplify all the above equations, we wish to note that there exists permutation symmetry between the *x* and *y* components for any physical quantities. For simplicity of notations in the following intermediate steps, we use $\lambda$ to replace the constant $\lambda\mathrm{Re}$ in the Helmholtz equation (I.7). The $\lambda\mathrm{Re}$ form will be restored in the final solution form.

Substituting (I.8) into (I.7), we obtain

$$\begin{matrix} -C_{2}k\left[\frac{d^{2}\tilde{u}_{z}(z)}{dz^{2}}+\left(\lambda-m^{2}-k^{2}\right)\tilde{u}_{z}(z)\right]-B_{1}\left[\frac{d^{3}\tilde{u}_{y}(z)}{dz^{3}}+\left(\lambda-m^{2}-k^{2}\right)\frac{d\tilde{u}_{y}(z)}{dz}\right]\text{=0} (I.12a) \\ C_{1}k\left[\frac{d^{2}\tilde{u}_{z}(z)}{dz^{2}}+\left(\lambda-m^{2}-k^{2}\right)\tilde{u}_{z}(z)\right]-B_{2}\left[\frac{d^{3}\tilde{u}_{y}(z)}{dz^{3}}+\left(\lambda-m^{2}-k^{2}\right)\frac{d\tilde{u}_{y}(z)}{dz}\right]\text{=0} (I.12b) \\ -C_{4}k\left[\frac{d^{2}\tilde{u}_{z}(z)}{dz^{2}}+\left(\lambda-m^{2}-k^{2}\right)\tilde{u}_{z}(z)\right]-B_{3}\left[\frac{d^{3}\tilde{u}_{y}(z)}{dz^{3}}+\left(\lambda-m^{2}-k^{2}\right)\frac{d\tilde{u}_{y}(z)}{dz}\right]\text{=0} (I.12c) \\ C_{3}k\left[\frac{d^{2}\tilde{u}_{z}(z)}{dz^{2}}+\left(\lambda-m^{2}-k^{2}\right)\tilde{u}_{z}(z)\right]-B_{4}\left[\frac{d^{3}\tilde{u}_{y}(z)}{dz^{3}}+\left(\lambda-m^{2}-k^{2}\right)\frac{d\tilde{u}_{y}(z)}{dz}\right]\text{=0} (I.12d) \end{matrix}$$

Substituting (9) into (7), we obtain

$$\begin{matrix} C_{3}m\left[\frac{d^{2}\tilde{u}_{z}(z)}{dz^{2}}+\left(\lambda-m^{2}-k^{2}\right)\tilde{u}_{z}(z)\right]+A_{1}\left[\frac{d^{3}\tilde{u}_{x}(z)}{dz^{3}}+\left(\lambda-m^{2}-k^{2}\right)\frac{d\tilde{u}_{x}(z)}{dz}\right]\text{=0} (I.13a) \\ C_{4}m\left[\frac{d^{2}\tilde{u}_{z}(z)}{dz^{2}}+\left(\lambda-m^{2}-k^{2}\right)\tilde{u}_{z}(z)\right]+A_{2}\left[\frac{d^{3}\tilde{u}_{x}(z)}{dz^{3}}+\left(\lambda-m^{2}-k^{2}\right)\frac{d\tilde{u}_{x}(z)}{dz}\right]\text{=0} (I.13b) \\ -C_{1}m\left[\frac{d^{2}\tilde{u}_{z}(z)}{dz^{2}}+\left(\lambda-m^{2}-k^{2}\right)\tilde{u}_{z}(z)\right]+A_{3}\left[\frac{d^{3}\tilde{u}_{x}(z)}{dz^{3}}+\left(\lambda-m^{2}-k^{2}\right)\frac{d\tilde{u}_{x}(z)}{dz}\right]\text{=0} (I.13c) \\ -C_{2}m\left[\frac{d^{2}\tilde{u}_{z}(z)}{dz^{2}}+\left(\lambda-m^{2}-k^{2}\right)\tilde{u}_{z}(z)\right]+A_{4}\left[\frac{d^{3}\tilde{u}_{x}(z)}{dz^{3}}+\left(\lambda-m^{2}-k^{2}\right)\frac{d\tilde{u}_{x}(z)}{dz}\right]\text{=0} (I.13d) \end{matrix}$$

Substituting (I.10) into (I.7) leads to

$$\begin{matrix} -B_{3}m\left[\frac{d^{2}\tilde{u}_{y}(z)}{dz^{2}}+\left(\lambda-m^{2}-k^{2}\right)\tilde{u}_{y}(z)\right]+A_{2}k\left[\frac{d^{2}\tilde{u}_{x}(z)}{dz^{2}}+\left(\lambda-m^{2}-k^{2}\right)\tilde{u}_{x}(z)\right]=0 (I.14a) \\ -B_{4}m\left[\frac{d^{2}\tilde{u}_{y}(z)}{dz^{2}}+\left(\lambda-m^{2}-k^{2}\right)\tilde{u}_{y}(z)\right]-A_{1}k\left[\frac{d^{2}\tilde{u}_{x}(z)}{dz^{2}}+\left(\lambda-m^{2}-k^{2}\right)\tilde{u}_{x}(z)\right]=0 (I.14b) \\ B_{1}m\left[\frac{d^{2}\tilde{u}_{y}(z)}{dz^{2}}+\left(\lambda-m^{2}-k^{2}\right)\tilde{u}_{y}(z)\right]+A_{4}k\left[\frac{d^{2}\tilde{u}_{x}(z)}{dz^{2}}+\left(\lambda-m^{2}-k^{2}\right)\tilde{u}_{x}(z)\right]=0 (I.14c) \\ B_{2}m\left[\frac{d^{2}\tilde{u}_{y}(z)}{dz^{2}}+\left(\lambda-m^{2}-k^{2}\right)\tilde{u}_{y}(z)\right]-A_{3}k\left[\frac{d^{2}\tilde{u}_{x}(z)}{dz^{2}}+\left(\lambda-m^{2}-k^{2}\right)\tilde{u}_{x}(z)\right]=0 (I.14d) \end{matrix}$$

The above sets of linear relations are the starting point for obtaining the dispersion relations of the hydrodynamic modes (HMs). In what follows we would like to show that among the 16 linear relations— (I.12a-d), (I.13a-d), (I.14a-d), and (I.3a-d), there are only 3 linearly independent equations, from which the HMs’ dispersion relations can be uniquely determined. We proceed by showing that only one out of the four equations in each of the above four sets of linear relations can be linearly independent. This is followed by showing that one set out of the three sets, (I.12a-d), (I.13a-d), and (I.14a-d), is redundant. In this manner the number of linearly independent relations is reduced to 3, from which the dispersion relation follows.

To reach the above goal, we would first like to show that$A_{3}B_{1}+A_{4}B_{2}=0$via proof by contradiction. $A_{3}B_{1}+A_{4}B_{2}=0$would mean that (I.14c) and (I.14d) are linearly dependent, while at the same time the left hand sides of (I.11c), (I.11d) are 0.

Suppose$A_{3}B_{1}+A_{4}B_{2}\neq0$. By using (I.11c) and (I.11d), we obtain

$\tilde{u}_{x}(z)=-\frac{1}{m}\frac{B_{1}C_{1}+B_{2}C_{2}}{A_{3}B_{1}+A_{4}B_{2}}\frac{d\tilde{u}_{z}(z)}{dz}$ (I.15a)

$\tilde{u}_{y}(z)=-\frac{1}{k}\frac{A_{3}C_{2}-A_{4}C_{1}}{A_{3}B_{1}+A_{4}B_{2}}\frac{d\tilde{u}_{z}(z)}{dz}$ (I.15b)

From this result, (I.12a-d) can be written as

$$\begin{matrix} -C_{2}k\left[\frac{d^{2}\tilde{u}_{z}(z)}{dz^{2}}+\left(\lambda-m^{2}-k^{2}\right)\tilde{u}_{z}(z)\right]+\frac{B_{1}}{k}\frac{A_{3}C_{2}-A_{4}C_{1}}{A_{3}B_{1}+A_{4}B_{2}}\left[\frac{d^{4}\tilde{u}_{z}(z)}{dz^{4}}+\left(\lambda-m^{2}-k^{2}\right)\frac{d^{2}\tilde{u}_{z}(z)}{dz^{2}}\right]\text{=0} (I.16a) \\ C_{1}k\left[\frac{d^{2}\tilde{u}_{z}(z)}{dz^{2}}+\left(\lambda-m^{2}-k^{2}\right)\tilde{u}_{z}(z)\right]+\frac{B_{2}}{k}\frac{A_{3}C_{2}-A_{4}C_{1}}{A_{3}B_{1}+A_{4}B_{2}}\left[\frac{d^{4}\tilde{u}_{z}(z)}{dz^{4}}+\left(\lambda-m^{2}-k^{2}\right)\frac{d^{2}\tilde{u}_{z}(z)}{dz^{2}}\right]\text{=0} (I.16b) \\ -C_{4}k\left[\frac{d^{2}\tilde{u}_{z}(z)}{dz^{2}}+\left(\lambda-m^{2}-k^{2}\right)\tilde{u}_{z}(z)\right]+\frac{B_{3}}{k}\frac{A_{3}C_{2}-A_{4}C_{1}}{A_{3}B_{1}+A_{4}B_{2}}\left[\frac{d^{4}\tilde{u}_{z}(z)}{dz^{4}}+\left(\lambda-m^{2}-k^{2}\right)\frac{d^{2}\tilde{u}_{z}(z)}{dz^{2}}\right]\text{=0} (I.16c) \\ C_{3}k\left[\frac{d^{2}\tilde{u}_{z}(z)}{dz^{2}}+\left(\lambda-m^{2}-k^{2}\right)\tilde{u}_{z}(z)\right]+\frac{B_{4}}{k}\frac{A_{3}C_{2}-A_{4}C_{1}}{A_{3}B_{1}+A_{4}B_{2}}\left[\frac{d^{4}\tilde{u}_{z}(z)}{dz^{4}}+\left(\lambda-m^{2}-k^{2}\right)\frac{d^{2}\tilde{u}_{z}(z)}{dz^{2}}\right]\text{=0} (I.16d) \end{matrix}$$

Equations (I.16a-d) possess the same algebraic structure. We choose (I.16a) as the focus of further analysis. We intend to show that the implications of (I.16a) lead to contradictions. Hence $A_{3}B_{1}+A_{4}B_{2}=0$must hold true.

To reach the above conclusion, we analyze the three possible cases for the numerator of the second term on the right hand side of (I.16a): ${\left( i \right) B}_{1}(A_{3}C_{2}-A_{4}C_{1})=0$, $C_{2}\neq0, {\left( ii \right) B}_{1}\left( A_{3}C_{2}-A_{4}C_{1} \right)=0,$ $C_{2}=0$ , and *(iii)* $B_{1}(A_{3}C_{2}-A_{4}C_{1})\neq0$

Case $\left( i \right):$ Since $C_{2}\neq0$, we have

$$\frac{d^{2}\tilde{u}_{z}(z)}{dz^{2}}+\left(\lambda-m^{2}-k^{2}\right)\tilde{u}_{z}(z)=0, \tilde{u}_{z}(\pm H)=0,$$

so that

$\tilde{u}_{z}(z)=\sin\left(\frac{n\pi}{H}z\right), n=0,1,2,...$ or $\tilde{u}_{z}(z)=cos\left(\frac{(2n+1)\pi}{2H}z\right), n=0,1,2,...$ (I.17a)

By substituting $\tilde{u}_{z}\left( z \right)$ into (I.15a) and (I.15b), we obtain

$\tilde{u}_{x}(z)=-\frac{1}{m}\frac{B_{1}C_{1}+B_{2}C_{2}}{A_{3}B_{1}+A_{4}B_{2}}\frac{n\pi}{H}\cos\left(\frac{n\pi}{H}z\right) or \frac{1}{m}\frac{B_{1}C_{1}+B_{2}C_{2}}{A_{3}B_{1}+A_{4}B_{2}}\frac{(2n+1)\pi}{2H}\sin\left(\frac{(2n+1)\pi}{2H}z\right)$ (I.17b)

$\tilde{u}_{y}(z)=-\frac{1}{k}\frac{A_{3}C_{2}-A_{4}C_{1}}{A_{3}B_{1}+A_{4}B_{2}}\frac{n\pi}{H}\cos\left(\frac{n\pi}{H}z\right) or \frac{1}{k}\frac{A_{3}C_{2}-A_{4}C_{1}}{A_{3}B_{1}+A_{4}B_{2}}\frac{(2n+1)\pi}{2H}\sin\left(\frac{(2n+1)\pi}{2H}z\right)$ (I.17c)

Even though the above expressions for $\tilde{u}_{x}\left( z \right), \tilde{u}_{y}\left( z \right)$ and $\tilde{u}_{z}(z)$ satisfy the vorticity (I.7), the incompressibility constraints (divergence free of velocity field) (I.3a-d), and the vanishing normal component of velocity $u_{z}(x,y,z)$, but if we substitue them back into (I.5, I.6), it is clear that the resulting dispersion relation $\lambda=\sqrt{\left( \frac{n\pi}{H} \right)^{2}+m^{2}+k^{2}}$ does not satisfy the Navier slip boundary condition. So $C_{2}\neq0$ is impossible.

Case *(ii)*: In this case $C_{2}=0$, and either $B_{1}=0$ or $A_{4}C_{1}=0$. From (I.3c) and (I.3d), it follows that either $\tilde{u}_{x}\left( z \right)=0$ or $\tilde{u}_{y}\left( z \right)=0$, or both. That contradicts our basic assumption of nonzero $\tilde{u}_{x}(z)$ and $\tilde{u}_{y}(z)$ components.

Case *(iii)*: In this case (I.16a) becomes a 4^th^ order homogeneous ODE and it has the general solution $\tilde{u}_{z}(z)\propto sin(\mu z)$. Solving for its characteristic equation we obtain

$\mu^{2}=\lambda-m^{2}-k^{2} \text{or} \mu^{2}=-\frac{C_{2}}{B_{1}}\frac{A_{3}B_{1}+A_{4}B_{2}}{A_{3}C_{2}-A_{4}C_{1}}k^{2}$.

Provided$A_{3}B_{1}+A_{4}B_{2}\neq0$, then from (I.14c,d) we must have

$\frac{d^{2}\tilde{u}_{x}(z)}{dz^{2}}+\left(\lambda-m^{2}-k^{2}\right)\tilde{u}_{x}(z)=0$ (I.18a)

$\frac{d^{2}\tilde{u}_{y}(z)}{dz^{2}}+\left(\lambda-m^{2}-k^{2}\right)\tilde{u}_{y}(z)=0$. (I.18b)

The linear relations (I.15a,b), combined with (I.18a,b), make it impossible to have the choice$\mu^{2}=-\frac{C_{2}}{B_{1}}\frac{A_{3}B_{1}+A_{4}B_{2}}{A_{3}C_{2}-A_{4}C_{1}}k^{2}$. In this manner there is only one remaining choice— $\mu^{2}=\lambda-m^{2}-k^{2}$. But it cannot simultaneously satisfy the vanishing normal component of velocity $u_{z}(x,y,z)$ and the Navier slip boundary condition, (I.5) and (I.6). This completes the proof by contradiction, hence $A_{3}B_{1}+A_{4}B_{2}=0$.

For the same reasons, we must have $A_{1}B_{3}+A_{2}B_{4}=0$. That means (I.14a,b) are also linearly dependent. As a result, we observe that the left-hand side coefficients of (I.11a-d) are all 0. Therefore either$\frac{d\tilde{u}_{z}(z)}{dz}=0$, or $B_{3}C_{3}+B_{4}C_{4}=A_{1}C_{4}-A_{2}C_{3}=B_{1}C_{1}+B_{2}C_{2}=A_{3}C_{2}-A_{4}C_{1}=0$. Since$\tilde{u}_{z}(z)\neq0$, we must have $\frac{d\tilde{u}_{z}(z)}{dz}\neq0$ since otherwise combined with the boundary condition—that the normal component must vanish at $z=\pm1$—would imply $\tilde{u}_{z}\left( z \right)=0$. Therefore we must have$B_{3}C_{3}+B_{4}C_{4}=A_{1}C_{4}-A_{2}C_{3}=B_{1}C_{1}+B_{2}C_{2}=A_{3}C_{2}-A_{4}C_{1}=0$. These four relations show that (I.12a,b), (I.12c,d), (I.13a,b), and (I.13c,d) are all linearly dependent.

Now the same process can be repeated for (I.14a,c) and (I.14b,d), to show that $A_{2}B_{1}+A_{4}B_{3}$=$A_{1}B_{2}+A_{3}B_{4}$= 0, which means all four (I.14a-d) are linearly dependent, and there is only one linearly independent relation.

Now we re-group (I.3a,c) and (I.3b,d) to obtain another set of similar equations

$$\begin{matrix} \left(A_{1}B_{2}+A_{3}B_{4}\right)m\tilde{u}_{x}(z)=-\left(B_{2}C_{3}-B_{4}C_{1}\right)\frac{d\tilde{u}_{z}(z)}{dz} (I.19a) \\ \left(A_{1}B_{2}+A_{3}B_{4}\right)k\tilde{u}_{y}(z)=\left(A_{1}C_{1}+A_{3}C_{3}\right)\frac{d\tilde{u}_{z}(z)}{dz} (I.19b) \\ \left(A_{2}B_{1}+A_{4}B_{3}\right)m\tilde{u}_{x}(z)=-\left(B_{1}C_{4}-B_{3}C_{2}\right)\frac{d\tilde{u}_{z}(z)}{dz} (I.19c) \\ \left(A_{2}B_{1}+A_{4}B_{3}\right)k\tilde{u}_{y}(z)=-\left(A_{2}C_{2}+A_{4}C_{4}\right)\frac{d\tilde{u}_{z}(z)}{dz} (I.19d) \end{matrix}$$

It can be seen that the left hand side of (I.19a-d) are all zero, Hence the following 6 coefficient relations can be obtained:

$\begin{matrix} A_{2}B_{1}+A_{4}B_{3}=A_{1}B_{2}+A_{3}B_{4}=-B_{2}C_{3}+B_{4}C_{1} \\ =A_{1}C_{1}+A_{3}C_{3}=B_{1}C_{4}-B_{3}C_{2}=A_{3}C_{2}-A_{4}C_{1} \\ =0 \end{matrix}$. (I.20)

Thus (I.12b) and (I.12d) ($-B_{2}C_{3}+B_{4}C_{1}=0$), and (I.13a) and (I.13c) ($A_{1}C_{1}+A_{3}C_{3}=0$) are also shown to be linearly dependent. As a result of the 12 such coefficient restrictions obtained above, the four equations in each of the three groups, (I.12a-d), (I.13a-d) and (I.14a-d), are all linearly dependent and there is only *one* linearly independent equation in each group. We choose (I.12a), (I.13a) and (I.14d) to form the following set of equations:

$$\begin{matrix} -B_{1}\left[\frac{d^{3}\tilde{u}_{y}(z)}{dz^{3}}+\left(\lambda-m^{2}-k^{2}\right)\frac{d\tilde{u}_{y}(z)}{dz}\right]-C_{2}k\left[\frac{d^{2}\tilde{u}_{z}(z)}{dz^{2}}+\left(\lambda-m^{2}-k^{2}\right)\tilde{u}_{z}(z)\right]\text{=0} (I.21a) \\ A_{1}\left[\frac{d^{3}\tilde{u}_{x}(z)}{dz^{3}}+\left(\lambda-m^{2}-k^{2}\right)\frac{d\tilde{u}_{x}(z)}{dz}\right]+C_{3}m\left[\frac{d^{2}\tilde{u}_{z}(z)}{dz^{2}}+\left(\lambda-m^{2}-k^{2}\right)\tilde{u}_{z}(z)\right]\text{=0} (I.21b) \\ -A_{3}k\left[\frac{d^{2}\tilde{u}_{x}(z)}{dz^{2}}+\left(\lambda-m^{2}-k^{2}\right)\tilde{u}_{x}(z)\right]+B_{2}m\left[\frac{d^{2}\tilde{u}_{y}(z)}{dz^{2}}+\left(\lambda-m^{2}-k^{2}\right)\tilde{u}_{y}(z)\right]=0 (I.21c) \end{matrix}$$

It is a general 3^rd^ order homogeneous ordinary differential equation for $\tilde{u}_{x}\left( z \right), \tilde{u}_{y}\left( z \right),$ and $\tilde{u}_{z}(z)$. By assuming  $\tilde{u}_{x\left( y,z \right)}(z)\propto e^{\mu z}$, where$\mu$may be regarded as the eigen-wavevector of the system, we have

$\left(\begin{matrix} 0 & -B_{1}\mu\left[\mu^{2}+\left(\lambda-m^{2}-k^{2}\right)\right] & -C_{2}k\left[\mu^{2}+\left(\lambda-m^{2}-k^{2}\right)\right] \\ -A_{3}k\left[\mu^{2}+\left(\lambda-m^{2}-k^{2}\right)\right] & B_{2}m\left[\mu^{2}+\left(\lambda-m^{2}-k^{2}\right)\right] & 0 \\ A_{1}\mu\left[\mu^{2}+\left(\lambda-m^{2}-k^{2}\right)\right] & 0 & C_{3}m\left[\mu^{2}+\left(\lambda-m^{2}-k^{2}\right)\right] \end{matrix}\right)\cdot\left(\begin{matrix} \tilde{u}_{x}(z) \\ \tilde{u}_{y}(z) \\ \tilde{u}_{z}(z) \end{matrix}\right)\text{=0}$. (I.22)

From (I.22), it is clear that at least one equation is redundant. Hence out of the three equations, (I.12a-c), we need only two.

Furthermore, by using $B_{3}C_{3}+B_{4}C_{4}=A_{1}C_{4}-A_{2}C_{3}=0$ we obtain that (I.3a,b) are linearly dependent; by using $A_{1}B_{2}+A_{3}B_{4}=A_{1}C_{1}+A_{3}C_{3}=0$, we obtain that (I.3a,c) are linearly dependent, and by using , we conclude that (I.3c,d) are also linearly dependent. Thus (I.3a-d) are also linearly dependent and we only need to select one out of the four equations.

Following the above discussion, we select (I.21a), (I.21c) and (I.3b) as the three linearly independent coefficients constraint relations:

$$\begin{matrix} -B_{1}\left[\frac{d^{3}\tilde{u}_{y}(z)}{dz^{3}}+\left(\lambda-m^{2}-k^{2}\right)\frac{d\tilde{u}_{y}(z)}{dz}\right]-C_{2}k\left[\frac{d^{2}\tilde{u}_{z}(z)}{dz^{2}}+\left(\lambda-m^{2}-k^{2}\right)\tilde{u}_{z}(z)\right]\text{=0} \\ -A_{3}k\left[\frac{d^{2}\tilde{u}_{x}(z)}{dz^{2}}+\left(\lambda-m^{2}-k^{2}\right)\tilde{u}_{x}(z)\right]+B_{2}m\left[\frac{d^{2}\tilde{u}_{y}(z)}{dz^{2}}+\left(\lambda-m^{2}-k^{2}\right)\tilde{u}_{y}(z)\right]=0 , (I.23) \\ A_{2}m\tilde{u}_{x}(z)+B_{3}k\tilde{u}_{y}(z)+C_{4}\frac{d\tilde{u}_{z}(z)}{dz}=0 \end{matrix}$$

or

$$\left(\begin{matrix} 0 & -B_{1}\mu\left[\mu^{2}+\left(\lambda-m^{2}-k^{2}\right)\right] & -C_{2}k\left[\mu^{2}+\left(\lambda-m^{2}-k^{2}\right)\right] \\ -A_{3}k\left[\mu^{2}+\left(\lambda-m^{2}-k^{2}\right)\right] & B_{2}m\left[\mu^{2}+\left(\lambda-m^{2}-k^{2}\right)\right] & 0 \\ A_{2}m & B_{3}k & C_{4}\mu\end{matrix}\right)\cdot\left(\begin{matrix} \tilde{u}_{x}(z) \\ \tilde{u}_{y}(z) \\ \tilde{u}_{z}(z) \end{matrix}\right)\text{=0} (I.24)$$

The condition for nonzero solutions for $\tilde{u}_{x}\left( z \right), \tilde{u}_{y}\left( z \right) and \tilde{u}_{z}(z)$ is that

$\left|\begin{matrix} 0 & -B_{1}\mu\left[\mu^{2}+\left(\lambda-m^{2}-k^{2}\right)\right] & -C_{2}k\left[\mu^{2}+\left(\lambda-m^{2}-k^{2}\right)\right] \\ -A_{3}k\left[\mu^{2}+\left(\lambda-m^{2}-k^{2}\right)\right] & B_{2}m\left[\mu^{2}+\left(\lambda-m^{2}-k^{2}\right)\right] & 0 \\ A_{2}m & B_{3}k & C_{4}\mu\end{matrix}\right|=0$ (I.25a)

By utilizing the derived coefficient relations, the determinant condition, (25a), can be simplified to be

$\left[\mu^{2}+\left(\lambda-m^{2}-k^{2}\right)\right]^{2}\left[-m^{2}+\mu^{2}-k^{2}\right]=0$ . (I.25b)

There are two roots for the eigen-wavevector $\mu$, $\mu^{(1)}=\pm i\sqrt{\lambda-m^{2}-k^{2}}$ and $\mu^{(2)}=\pm\sqrt{m^{2}+k^{2}}$. Since the velocity field is a real variable, therefore for convenience we divide the basis functions $e^{\mu^{(1)}z}$, $e^{\mu^{(2)}z}$ into two cases based on their symmetry properties.

**Anti-symmetric case:**

$\begin{matrix} \tilde{u}_{x}(z)=u_{1}\sin(\sqrt{\lambda-m^{2}-k^{2}}z)+u_{2}\sinh(\sqrt{m^{2}+k^{2}}z) \\ \tilde{u}_{y}(z)=v_{1}\sin(\sqrt{\lambda-m^{2}-k^{2}}z)+v_{2}\sinh(\sqrt{m^{2}+k^{2}}z) \\ \tilde{u}_{z}(z)=w_{1}\cos(\sqrt{\lambda-m^{2}-k^{2}}z)+w_{2}\cosh(\sqrt{m^{2}+k^{2}}z) \end{matrix}$ (I.26)

By substituting (I.26) into (I.23), we obtain

$\begin{matrix} -B_{1}\lambda\sqrt{m^{2}+k^{2}}v_{2}-C_{2}k\lambda w_{2}\text{=0} \\ -A_{3}k\lambda u_{2}+B_{2}m\lambda v_{2}=0 \\ A_{2}mu_{2}+B_{3}kv_{2}+C_{4}\sqrt{m^{2}+k^{2}}w_{2}=0 \\ A_{2}mu_{1}+B_{3}kv_{1}-C_{4}\sqrt{\lambda-m^{2}-k^{2}}w_{1}=0 \end{matrix}$ (I.27)

The first 3 equations are linearly dependent according to (I.24). Therefore we choose to neglect the third one. Equation (I.27), together with the boundary conditions, (I.4- I.6), means that the following three conditions must hold:

$\begin{matrix} \left[l_{s}\sqrt{\lambda-m^{2}-k^{2}}\cos(\sqrt{\lambda-m^{2}-k^{2}})+\sin(\sqrt{\lambda-m^{2}-k^{2}})\right]u_{1} \\ +\left[l_{s}\sqrt{m^{2}+k^{2}}\cosh(\sqrt{m^{2}+k^{2}})+\sinh(\sqrt{m^{2}+k^{2}})\right]u_{2}=0 \end{matrix}$ (I.28a)

$\begin{matrix} \left[l_{s}\sqrt{\lambda-m^{2}-k^{2}}\cos(\sqrt{\lambda-m^{2}-k^{2}})+\sin(\sqrt{\lambda-m^{2}-k^{2}})\right]v_{1} \\ +\left[l_{s}\sqrt{m^{2}+k^{2}}\cosh(\sqrt{m^{2}+k^{2}})+\sinh(\sqrt{m^{2}+k^{2}})\right]v_{2}=0 \end{matrix}$ (I.28b)

$\cos(\sqrt{\lambda-m^{2}-k^{2}})w_{1}+\cosh(\sqrt{m^{2}+k^{2}})w_{2}=0$ (I.28c)

For simplicity, in what follows we denote $\mu=\sqrt{\lambda-m^{2}-k^{2}}$ and $\nu=\sqrt{m^{2}+k^{2}}$. Now there are 6 un-determined coefficients and 6 constraint equations; they can be expressed in the following matrix form:

The determinant of the coefficients matrix should be 0 in order to have nonzero solutions. By using the block matrix formula $\left|\begin{matrix} A & B \\ C & D \end{matrix}\right|=\left|A-BD^{-1}C\right|\cdot\left|D\right|$ to compute the above 6 by 6 determinant, each block matrix is a 3 by 3 submatrix. Notice that $l_{s}\nu\cosh(\nu)+\sinh(\nu)$ is always greater than 0 for positive $\nu$ (consider the function $f\left( x \right)=l_{s}x+\tanh\left( x \right),$ with *f*(0)=0 and $f^{'}\left( x \right)>0$), thus

$$\det\left(M\right)=0\Leftrightarrow\left|A-BD^{-1}C\right|=0$$

$$A=\left(\begin{matrix} A_{2}m & B_{3}k & -C_{4}\mu\\ 0 & 0 & 0 \\ 0 & 0 & 0 \end{matrix}\right), B=\left(\begin{matrix} 0 & 0 & 0 \\ 0 & B_{1}\lambda\nu& C_{2}k\lambda\\ -A_{3}k\lambda& B_{2}m\lambda& 0 \end{matrix}\right)$$

$$C=\left(\begin{matrix} l_{s}\mu\cos(\mu)+\sin(\mu) & 0 & 0 \\ 0 & l_{s}\mu\cos(\mu)+\sin(\mu) & 0 \\ 0 & 0 & \cos(\mu) \end{matrix}\right)$$

$$\begin{matrix} D^{-1}=\left(\begin{matrix} \frac{1}{l_{s}\nu\cosh(\nu)+\sinh(\nu)} & 0 & 0 \\ 0 & \frac{1}{l_{s}\nu\cosh(\nu)+\sinh(\nu)} & 0 \\ 0 & 0 & \frac{1}{\cosh(\nu)} \end{matrix}\right) \\ \left(A-BD^{-1}C\right)=\left(\begin{matrix} A_{2}m & B_{3}k & -C_{4}\mu\\ 0 & -\frac{l_{s}\mu\cos(\mu)+\sin(\mu)}{l_{s}\nu\cosh(\nu)+\sinh(\nu)}B_{1}\lambda\nu& -\frac{\cos(\mu)}{\cosh(\nu)}C_{2}k\lambda\\ \frac{l_{s}\mu\cos(\mu)+\sin(\mu)}{l_{s}\nu\cosh(\nu)+\sinh(\nu)}A_{3}k\lambda& -\frac{l_{s}\mu\cos(\mu)+\sin(\mu)}{l_{s}\nu\cosh(\nu)+\sinh(\nu)}B_{2}m\lambda& 0 \end{matrix}\right) \end{matrix}$$

$\begin{matrix} \det\left(A-BD^{-1}C\right)=-A_{2}B_{2}C_{2}m^{2}k\lambda^{2}\frac{l_{s}\mu\cos(\mu)+\sin(\mu)}{l_{s}\nu\cosh(\nu)+\sinh(\nu)}\frac{\cos(\mu)}{\cosh(\nu)} \\ +A_{3}k\lambda\frac{l_{s}\mu\cos(\mu)+\sin(\mu)}{l_{s}\nu\cosh(\nu)+\sinh(\nu)}\left[-B_{3}C_{2}k^{2}\lambda\frac{\cos(\mu)}{\cosh(\nu)}-\frac{l_{s}\mu\cos(\mu)+\sin(\mu)}{l_{s}\nu\cosh(\nu)+\sinh(\nu)}B_{1}C_{4}\lambda\nu\mu\right] \end{matrix}$ (I.29)

Hence $\det\left(A-BD^{-1}C\right)=0$ is equivalent to

(I.30)

By using the coefficients relations, (I.20), we obtain

$$A_{3}C_{2}-A_{4}C_{1}=0 \to\left(A_{3}C_{2}-A_{4}C_{1}\right)B_{3}=0 \to A_{3}C_{2}B_{3}=A_{4}C_{1}B_{3}$$

$$\begin{matrix} A_{4}B_{3}+A_{2}B_{1}=0 \to\left(A_{4}B_{3}+A_{2}B_{1}\right)C_{1}=0 \to A_{4}C_{1}B_{3}=-A_{2}B_{1}C_{1} \\ etc. \end{matrix}$$

In this manner, we obtain the following relations:

$A_{3}B_{3}C_{2}=A_{4}C_{1}B_{3}=-A_{2}B_{1}C_{1}=A_{2}B_{2}C_{2}, A_{3}B_{1}C_{4}=-A_{4}B_{2}C_{4}=A_{2}C_{2}B_{2}$.

These relations enable us to reduce (I.30) to the following form:

$-A_{2}B_{2}C_{2}\frac{l_{s}\mu\cos(\mu)+\sin(\mu)}{l_{s}\nu\cosh(\nu)+\sinh(\nu)}\frac{\cos(\mu)}{\cosh(\nu)}\nu k\lambda^{2}\left[\nu+\mu\frac{l_{s}\mu+\tan(\mu)}{l_{s}\nu+\tanh(\nu)}\right]=0$ . (I.31)

Hence the dispersion relation is given by

$\nu[l_{s}\nu+\tanh(\nu)]+\mu[l_{s}\mu+\tan(\mu)]=0$ (I.32)

for the antisymmetric case. The corresponding coefficients can be easily solved in terms of$u_{1}$:

(I.33)

Without loss of generality, we can set $u_{1}=1$. Then

$\begin{matrix} \tilde{u}_{x}(z)=\sin(\sqrt{\lambda-m^{2}-k^{2}}z)-\frac{l_{s}\mu\cos(\mu)+\sin(\mu)}{l_{s}\nu\cosh(\nu)+\sinh(\nu)}\sinh(\nu z) \\ \tilde{u}_{y}(z)=\frac{A_{3}}{B_{2}}\frac{k}{m}\sin(\sqrt{\lambda-m^{2}-k^{2}}z)-\frac{A_{3}}{B_{2}}\frac{k}{m}\frac{l_{s}\mu\cos(\mu)+\sin(\mu)}{l_{s}\nu\cosh(\nu)+\sinh(\nu)}\sinh(\nu z) \\ \tilde{u}_{z}(z)=\frac{A_{3}}{C_{1}}\frac{\nu}{m}\frac{l_{s}\mu\cos(\mu)+\sin(\mu)}{l_{s}\nu\cosh(\nu)+\sinh(\nu)}\left[\frac{\cosh(\nu)}{\cos(\mu)}\cos(\sqrt{\lambda-m^{2}-k^{2}}z)-\cosh(\nu z)\right] \end{matrix}$ (I.34)

Now we replace $\lambda$ by $\lambda\mathrm{Re}$ back into (I.34); the HMs of the 3D incompressible (linearized) Navier-Stokes equation are given by:

$$\begin{matrix} u_{x}=\left[\sin(\sqrt{\lambda\mathrm{Re}-m^{2}-k^{2}}z)-\frac{l_{s}\mu\cos(\mu)+\sin(\mu)}{l_{s}\nu\cosh(\nu)+\sinh(\nu)}\sinh(\nu z)\right] \\ \cdot\left[A_{1}\sin(mx)\sin(ky)+A_{2}\sin(mx)\cos(ky)+A_{3}\cos(mx)\sin(ky)+A_{4}\cos(mx)\cos(ky)\right] \\ u_{y}=\frac{k}{m}\left[\sin(\sqrt{\lambda\mathrm{Re}-m^{2}-k^{2}}z)-\frac{l_{s}\mu\cos(\mu)+\sin(\mu)}{l_{s}\nu\cosh(\nu)+\sinh(\nu)}\sinh(\nu z)\right] (I.35) \\ \cdot\left[-A_{4}\sin(mx)\sin(ky)+A_{3}\sin(mx)\cos(ky)+A_{2}\cos(mx)\sin(ky)-A_{1}\cos(mx)\cos(ky)\right] \\ u_{z}=\frac{\nu}{m}\frac{l_{s}\mu\cos(\mu)+\sin(\mu)}{l_{s}\nu\cosh(\nu)+\sinh(\nu)}\left[\frac{\cosh(\nu)}{\cos(\mu)}\cos(\sqrt{\lambda\mathrm{Re}-m^{2}-k^{2}}z)-\cosh(\nu z)\right] \\ \cdot\left[A_{3}\sin(mx)\sin(ky)+A_{4}\sin(mx)\cos(ky)-A_{1}\cos(mx)\sin(ky)-A_{2}\cos(mx)\cos(ky)\right] \end{matrix}$$

$A_{1},A_{2},A_{3},A_{4}$ are four independent parameters used for adjusting the phase of the velocity modes in the *xy* plane. It reflects the fact that under the Navier slip boundary condition with constant slip length, the eigenmodes have translational symmetry in the $xy$ plane. By letting $A_{1}=1$ and $A_{2}=A_{3}=A_{4}=0$ , we have

$\begin{matrix} u_{x}=\left[\sin(\sqrt{\lambda\mathrm{Re}-m^{2}-k^{2}}z)-\frac{l_{s}\mu\cos(\mu)+\sin(\mu)}{l_{s}\nu\cosh(\nu)+\sinh(\nu)}\sinh(\nu z)\right]\sin(mx)\sin(ky) \\ u_{y}=-\frac{k}{m}\left[\sin(\sqrt{\lambda\mathrm{Re}-m^{2}-k^{2}}z)-\frac{l_{s}\mu\cos(\mu)+\sin(\mu)}{l_{s}\nu\cosh(\nu)+\sinh(\nu)}\sinh(\nu z)\right]\cos(mx)\cos(ky) \\ u_{z}=-\frac{\nu}{m}\frac{l_{s}\mu\cos(\mu)+\sin(\mu)}{l_{s}\nu\cosh(\nu)+\sinh(\nu)}\left[\frac{\cosh(\nu)}{\cos(\mu)}\cos(\sqrt{\lambda\mathrm{Re}-m^{2}-k^{2}}z)-\cosh(\nu z)\right]\cos(mx)\sin(ky) \end{matrix}$ (I.36a)

For completeness, we also write down the pressure expession associated with the above HM by solving a pressure Laplace equation$\nabla^{2}p=0$with the boundary condition $\left\{\partial p/\partial z=(\mathrm{Re})^{-1}\partial^{2}u_{z}/\partial z^{2}\right\}_{z=\pm1}$:

$p=\frac{\lambda}{m}\frac{l_{s}\mu\cos(\mu)+\sin(\mu)}{l_{s}\nu\cosh(\nu)+\sinh(\nu)}\sinh\left(\nu z\right)\cos(mx)\sin(ky)$ . (I.36b)

**Symmetric case:**

$\begin{matrix} \tilde{u}_{x}(z)=u'_{1}\cos(\sqrt{\lambda-m^{2}-k^{2}}z)+u'_{2}\cosh(\nu z) \\ \tilde{u}_{y}(z)=v'_{1}\cos(\sqrt{\lambda-m^{2}-k^{2}}z)+v'_{2}\cosh(\nu z) \\ \tilde{u}_{z}(z)=w'_{1}\sin(\sqrt{\lambda-m^{2}-k^{2}}z)+w'_{2}\sinh(\nu z) \end{matrix}$ (I.37)

$\begin{matrix} -B_{1}\lambda\sqrt{m^{2}+k^{2}}v'_{2}-C_{2}k\lambda w'_{2}\text{=0} \\ -A_{3}k\lambda u'_{2}+B_{2}m\lambda v'_{2}=0 \\ A_{2}mu'_{2}+B_{3}kv'_{2}+C_{4}\sqrt{m^{2}+k^{2}}w'_{2}=0 \\ A_{2}mu'_{1}+B_{3}kv'_{1}+C_{4}\sqrt{\lambda-m^{2}-k^{2}}w'_{1}=0 \end{matrix}$ (I.38)

Following the same procedure, and combining with the boundary conditions (I.4- I.6), we get (by choosing to neglect the third equation in (I.38), since one of them is redundant)

By using the same block matrix formula with

$\begin{matrix} A=\left(\begin{matrix} A_{2}m & B_{3}k & C_{4}\mu\\ 0 & 0 & 0 \\ 0 & 0 & 0 \end{matrix}\right), B=\left(\begin{matrix} 0 & 0 & 0 \\ 0 & B_{1}\lambda\nu& C_{2}k\lambda\\ -A_{3}k\lambda& B_{2}m\lambda& 0 \end{matrix}\right) \\ D^{-1}=\left(\begin{matrix} \frac{1}{-l_{s}\nu\sinh(\nu)-\cosh(\nu)} & 0 & 0 \\ 0 & \frac{1}{-l_{s}\nu\sinh(\nu)-\cosh(\nu)} & 0 \\ 0 & 0 & \frac{1}{\sinh(\nu)} \end{matrix}\right) \\ C=\left(\begin{matrix} l_{s}\mu\sin(\mu)-\cos(\mu) & 0 & 0 \\ 0 & l_{s}\mu\sin(\mu)-\cos(\mu) & 0 \\ 0 & 0 & \sin(\mu) \end{matrix}\right) \end{matrix}$ (I.39)

$A-BD^{-1}C=\left(\begin{matrix} A_{2}m & B_{3}k & C_{4}\mu\\ 0 & \frac{l_{s}\mu\sin(\mu)-\cos(\mu)}{l_{s}\nu\sinh(\nu)+\cosh(\nu)}B_{1}\lambda\nu& -\frac{\sin(\mu)}{\sinh(\nu)}C_{2}k\lambda\\ -\frac{l_{s}\mu\sin(\mu)-\cos(\mu)}{l_{s}\nu\sinh(\nu)+\cosh(\nu)}A_{3}k\lambda& \frac{l_{s}\mu\sin(\mu)-\cos(\mu)}{l_{s}\nu\sinh(\nu)+\cosh(\nu)}B_{2}m\lambda& 0 \end{matrix}\right)$ (I.40)

The condition that the determinant of the above matrix must be zero leads to the following equation:

$\begin{matrix} \left|A-BD^{-1}C\right|=A_{2}B_{2}C_{2}m^{2}k\lambda^{2}\frac{l_{s}\mu\sin(\mu)-\cos(\mu)}{l_{s}\nu\sinh(\nu)+\cosh(\nu)}\frac{\sin(\mu)}{\sinh(\nu)} \\ -\frac{l_{s}\mu\sin(\mu)-\cos(\mu)}{l_{s}\nu\sinh(\nu)+\cosh(\nu)}A_{3}k\lambda\left[-B_{3}C_{2}k^{2}\lambda\frac{\sin(\mu)}{\sinh(\nu)}-C_{4}B_{1}\lambda\mu\nu\frac{l_{s}\mu\sin(\mu)-\cos(\mu)}{l_{s}\nu\sinh(\nu)+\cosh(\nu)}\right] \\ =A_{2}B_{2}C_{2}\frac{l_{s}\mu\sin(\mu)-\cos(\mu)}{l_{s}\nu\sinh(\nu)+\cosh(\nu)}\frac{\sin(\mu)}{\sinh(\nu)}k\lambda^{2}\nu\left[\nu+\mu\frac{l_{s}\mu-\frac{1}{\tan(\mu)}}{l_{s}\nu+\frac{1}{\tanh(\nu)}}\right]=0 \end{matrix}$ (I.41)

Hence the dispersion relation for the symmetric case is given by

$\nu\left[l_{s}\nu+\frac{1}{\tanh(\nu)}\right]+\mu\left[l_{s}\mu+\frac{1}{\tan(\mu)}\right]=0$ (I.42)

for the symmetric case. In the same way we derive the corresponding coefficients

$\begin{matrix} u_{2}'=\frac{l_{s}\mu\sin(\mu)-\cos(\mu)}{l_{s}\nu\sinh(\nu)+\cosh(\nu)}u_{1}' \\ v_{2}'=\frac{A_{3}k}{B_{2}m}u_{2}'=\frac{A_{3}k}{B_{2}m}\frac{l_{s}\mu\sin(\mu)-\cos(\mu)}{l_{s}\nu\sinh(\nu)+\cosh(\nu)}u_{1}' \\ w_{2}'=-\frac{B_{1}\nu}{C_{2}k}v_{2}'=-\frac{B_{1}A_{3}}{C_{2}B_{2}}\frac{\nu}{m}\frac{l_{s}\mu\sin(\mu)-\cos(\mu)}{l_{s}\nu\sinh(\nu)+\cosh(\nu)}u_{1}'=\frac{A_{4}}{C_{2}}\frac{\nu}{m}\frac{l_{s}\mu\sin(\mu)-\cos(\mu)}{l_{s}\nu\sinh(\nu)+\cosh(\nu)}u_{1}' \\ v_{1}'=\frac{l_{s}\nu\sinh(\nu)+\cosh(\nu)}{l_{s}\mu\sin(\mu)-\cos(\mu)}v_{2}'=\frac{A_{3}k}{B_{2}m}u_{1}' \\ w_{1}'=-\frac{\sinh(\nu)}{\sin(\mu)}w_{2}'=-\frac{A_{4}}{C_{2}}\frac{\nu}{m}\frac{\sinh(\gamma)}{\sin(\mu)}\frac{l_{s}\mu\sin(\mu)-\cos(\mu)}{l_{s}\nu\sinh(\nu)+\cosh(\nu)}u_{1}' \end{matrix}$ (I.43)

At last, we can write down the velocity eigenfunctions explicitly as (without loss of generality we set ${u'}_{1}=1$ and replace $\lambda$ by $\lambda\mathrm{Re}$)

(I.44)

By letting $A_{1}=1$ and $A_{2}=A_{3}=A_{4}=0$ , (I.44) can be re-written as

(I.45a)

The accompanying pressure expression for the above HM is given by solving the pressure Laplace equation similar to the antisymmetric case:

$p=-\frac{\lambda}{m}\frac{l_{s}\mu\sin(\mu)-\cos(\mu)}{l_{s}\nu\sinh(\nu)+\cosh(\nu)}\cosh(\nu z)\cos(mx)\sin(ky)$ . (I.45b)

**Solution verification**

1. ***Incompressibility condition***

*The symmetric case:*

$$\begin{matrix} \nabla\cdot u=\left[m\cos(\mu z)+m\frac{l_{s}\mu\sin(\mu)-\cos(\mu)}{l_{s}\nu\sinh(\nu)+\cosh(\nu)}\cosh(\nu z)\right]\cos(mx)\sin(ky) \\ +\left[\frac{k^{2}}{m}\cos(\mu z)+\frac{k^{2}}{m}\frac{l_{s}\mu\sin(\mu)-\cos(\mu)}{l_{s}\nu\sinh(\nu)+\cosh(\nu)}\cosh(\nu z)\right]\cos(mx)\sin(ky) \\ +\left[\frac{\nu\mu}{m}\frac{\sinh(\nu)}{\sin(\mu)}\frac{l_{s}\mu\sin(\mu)-\cos(\mu)}{l_{s}\nu\sinh(\nu)+\cosh(\nu)}\cos(\mu z)-\frac{\nu^{2}}{m}\frac{l_{s}\mu\sin(\mu)-\cos(\mu)}{l_{s}\nu\sinh(\nu)+\cosh(\nu)}\cosh(\nu z)\right]\cos(mx)\sin(ky) \\ =\left[m+\frac{k^{2}}{m}+\frac{\nu\mu}{m}\frac{\sinh(\nu)}{\sin(\mu)}\frac{l_{s}\mu\sin(\mu)-\cos(\mu)}{l_{s}\nu\sinh(\nu)+\cosh(\nu)}\right]\cos(\mu z)\cos(mx)\sin(ky) \\ +\left[\left(m+\frac{k^{2}}{m}-\frac{\nu^{2}}{m}\right)\frac{l_{s}\mu\sin(\mu)-\cos(\mu)}{l_{s}\nu\sinh(\nu)+\cosh(\nu)}\right]\cosh(\nu z)\cos(mx)\sin(ky) \\ =\left[m+\frac{k^{2}}{m}-\frac{\nu^{2}}{m}\right]\cos(\mu z)\cos(mx)\sin(ky)+0 \\ \equiv0 \end{matrix}$$

In the above we have used the dispersion relation, (I.42), to simplify the following expression:

$$\frac{\sinh(\nu)}{\sin(\mu)}\frac{l_{s}\mu\sin(\mu)-\cos(\mu)}{l_{s}\nu\sinh(\nu)+\cosh(\nu)}=\frac{l_{s}\mu-\cot\left(\mu\right)}{l_{s}\nu+\coth\left(\nu\right)}=-\frac{\nu}{\mu}, \nu^{2}=m^{2}+k^{2}$$

*The antisymmetric case*

$$\begin{matrix} \nabla\cdot u=\left[m\sin(\sqrt{\lambda\mathrm{Re}-m^{2}-k^{2}}z)-m\frac{l_{s}\mu\cos(\mu)+\sin(\mu)}{l_{s}\nu\cosh(\nu)+\sinh(\nu)}\sinh(\sqrt{m^{2}+k^{2}}z)\right]\cos(mx)\sin(ky) \\ +\frac{k^{2}}{m}\left[\sin(\sqrt{\lambda\mathrm{Re}-m^{2}-k^{2}}z)-\frac{l_{s}\mu\cos(\mu)+\sin(\mu)}{l_{s}\nu\cosh(\nu)+\sinh(\nu)}\sinh(\sqrt{m^{2}+k^{2}}z)\right]\cos(mx)\sin(ky) \\ -\frac{\nu}{m}\frac{l_{s}\mu\cos(\mu H)+\sin(\mu)}{l_{s}\nu\cosh(\nu)+\sinh(\nu)}\left[-\mu\frac{\cosh(\gamma)}{\cos(\mu)}\sin(\sqrt{\lambda\mathrm{Re}-m^{2}-k^{2}}z)-v\sinh(vz)\right]\cos(mx)\sin(ky) \\ =\left[m+\frac{k^{2}}{m}+\frac{\mu\nu}{m}\frac{\cosh(\gamma)}{\cos(\mu)}\frac{l_{s}\mu\cos(\mu H)+\sin(\mu)}{l_{s}\nu\cosh(\nu)+\sinh(\nu)}\right]\sin\left(\mu z\right)\cos\left(mx\right)\sin\left(ky\right) \\ +\left[-m-\frac{k^{2}}{m}+\frac{\nu^{2}}{m}\right]\frac{l_{s}\mu\cos(\mu)+\sin(\mu)}{l_{s}\nu\cosh(\nu)+\sinh(\nu)}\sinh(vz)\cos(mx)\sin(ky) \\ =\left[m+\frac{k^{2}}{m}-\frac{\nu^{2}}{m}\right]\sin\left(\mu z\right)\cos\left(mx\right)\sin\left(ky\right)+0 \\ =0 \end{matrix}$$

In the above we have used the dispersion relation, (I.32), to simplify the following expression:

$$\frac{\cosh(\gamma)}{\cos(\mu)}\frac{l_{s}\mu\cos(\mu H)+\sin(\mu)}{l_{s}\nu\cosh(\nu)+\sinh(\nu)}=\frac{l_{s}\mu+\tan(\mu)}{l_{s}\nu+\tanh(\nu)}=-\frac{\nu}{\mu}$$

1. $u_{z}\boldsymbol{=0}$ ***at*** $\boldsymbol{z=\pm1}$

*The symmetric case*

$$\begin{matrix} u_{z}(x,y,\pm1)=\left[\pm\frac{\nu}{m}\frac{\sinh(\nu)}{\sin(\mu)}\frac{l_{s}\mu\sin(\mu)-\cos(\mu)}{l_{s}\nu\sinh(\nu)+\cosh(\nu)}\sin(\mu)\mp\frac{\nu}{m}\frac{l_{s}\mu\sin(\mu)-\cos(\mu)}{l_{s}\nu\sinh(\nu)+\cosh(\nu)}\sinh(\nu)\right]\cos(mx)\sin(ky) \\ =\left[\pm\frac{\nu}{m}\frac{l_{s}\mu\sin(\mu)-\cos(\mu)}{l_{s}\nu\sinh(\nu)+\cosh(\nu)}\sinh(\nu)\mp\frac{\nu}{m}\frac{l_{s}\mu\sin(\mu)-\cos(\mu)}{l_{s}\nu\sinh(\nu)+\cosh(\nu)}\sinh(\nu)\right]\cos(mx)\sin(ky)=0 \end{matrix}$$

*The antisymmetric case*

$$u_{z}\left(x,y,\pm1\right)=-\frac{\nu}{m}\frac{l_{s}\mu\cos(\mu)+\sin(\mu)}{l_{s}\nu\cosh(\nu)+\sinh(\nu)}\left[\frac{\cosh(v)}{\cos(\mu)}\cos(\mu)-\cosh(\nu)\right]\cos(mx)\sin(ky)=0$$

1. ***Navier slip boundary condition***

*The symmetric case*

*The antisymmetric case*

1. ***Navier Stokes eigenfunctions***

*The symmetric case*

We use $A_{1}=1$ branch for example, the other branches could be similarly verified.

$$\begin{matrix} -\nabla p+\frac{1}{\mathrm{Re}}\nabla^{2}u_{x}=-\lambda\frac{l_{s}\mu\sin(\mu)-\cos(\mu)}{l_{s}\nu\sinh(\nu)+\cosh(\nu)}\cosh(\nu z)\sin(mx)\sin(ky)-\frac{1}{\mathrm{Re}}\lambda\mathrm{Resin}(mx)\sin(ky)\cos(\sqrt{\lambda\mathrm{Re}-m^{2}-k^{2}}z) \\ =-\lambda\left[\cos(\sqrt{\lambda\mathrm{Re}-m^{2}-k^{2}}z)+\frac{l_{s}\mu\sin(\mu)-\cos(\mu)}{l_{s}\nu\sinh(\nu)+\cosh(\nu)}\cosh(\nu z)\right]\sin(mx)\sin(ky) \\ =-\lambda u_{x} \end{matrix}$$

*The antisymmetric case*

We use $u_{x}$ component of the $A_{1}=1$ branch for example, the other branches could be similarly verified.

$$\begin{matrix} -\nabla p+\frac{1}{\mathrm{Re}}\nabla^{2}u_{x}=\lambda\frac{l_{s}\mu\cos(\mu)+\sin(\mu)}{l_{s}\nu\cosh(\nu)+\sinh(\nu)}\sinh\left(\nu z\right)\sin(mx)\sin(ky)-\frac{1}{\mathrm{Re}}\lambda\mathrm{Resin}(\sqrt{\lambda\mathrm{Re}-m^{2}-k^{2}}z)\sin(mx)\sin(ky) \\ =-\lambda\left[\sin\left(\nu z\right)-\frac{l_{s}\mu\cos(\mu)+\sin(\mu)}{l_{s}\nu\cosh(\nu)+\sinh(\nu)}\sinh\left(\nu z\right)\right]\sin(mx)\sin(ky) \\ =-\lambda u_{x} \end{matrix}$$

Hence the 3D HMs satisfy all the boundary conditions. Together with their completeness and orthogonal properties, the HMs are therefore the perfect basis set for expanding the velocity ***u*** in the channel geometry.

## II. Unified expressions for the hydrodynamic modes

The HMs are orthonomal and complete eigenfunctions of the Navier Stokes equations in the channel geometry. In part A we have described the derivation of the 3D HMs in the channel flow geometry with the Navier slip boundary condition imposed at the upper and

lower solid walls. In previous publications, the 1D and 2D HMs were also derived. Here we rewrite them into one unified expression.

We first summarize the 2D and 3D HMs of the dimensionless Navier Stokes equation as follows. It should be noted that the 2D HMs can be obtained from the 3D solutions by setting $m=0,u_{x}=0$or $k=0,u_{y}=0$.

**Antisymmetric Case**

$\begin{matrix} u_{x}=m\left[\sin(\mu z)-\frac{l_{s}\mu\cos(\mu)+\sin(\mu)}{l_{s}\nu\cosh(\nu)+\sinh(\nu)}\sinh(\nu z)\right] \\ \cdot\left[A_{1}\sin(mx)\sin(ky)+A_{2}\sin(mx)\cos(ky)+A_{3}\cos(mx)\sin(ky)+A_{4}\cos(mx)\cos(ky)\right] \\ u_{y}=k\left[\sin(\mu z)-\frac{l_{s}\mu\cos(\mu)+\sin(\mu)}{l_{s}\nu\cosh(\nu)+\sinh(\nu)}\sinh(\nu z)\right] \\ \cdot\left[-A_{4}\sin(mx)\sin(ky)+A_{3}\sin(mx)\cos(ky)+A_{2}\cos(mx)\sin(ky)-A_{1}\cos(mx)\cos(ky)\right] \\ u_{z}=\gamma\frac{l_{s}\mu\cos(\mu)+\sin(\mu)}{l_{s}\nu\cosh(\nu)+\sinh(\nu)}\left[\frac{\cosh(\nu)}{\cos(\mu)}\cos(\mu z)-\cosh(\nu z)\right] \\ \cdot\left[A_{3}\sin(mx)\sin(ky)+A_{4}\sin(mx)\cos(ky)-A_{1}\cos(mx)\sin(ky)-A_{2}\cos(mx)\cos(ky)\right] \end{matrix}$ (II.1)

$\mu=\sqrt{\lambda\mathrm{Re}-m^{2}-k^{2}},\nu=\sqrt{m^{2}+k^{2}}$

$$\nu\left[l_{s}\nu+\tanh(\nu)\right]+\mu\left[l_{s}\mu+\tan(\mu)\right]=0$$

Here $m,k$ cannot both be 0 at the same time.

**Symmetric Case**

$\begin{matrix} u_{x}=m\left[\cos(\mu z)+\frac{l_{s}\mu\sin(\mu)-\cos(\mu)}{l_{s}\nu\sinh(\nu)+\cosh(\nu)}\cosh(\nu z)\right] \\ \cdot\left[A_{1}\sin(mx)\sin(ky)+A_{2}\sin(mx)\cos(ky)+A_{3}\cos(mx)\sin(ky)+A_{4}\cos(mx)\cos(ky)\right] \\ u_{y}=k\left[\cos(\mu z)+\frac{l_{s}\mu\sin(\mu)-\cos(\mu)}{l_{s}\nu\sinh(\nu)+\cosh(\nu)}\cosh(\nu z)\right] \\ \cdot\left[-A_{4}\sin(mx)\sin(ky)+A_{3}\sin(mx)\cos(ky)+A_{2}\cos(mx)\sin(ky)-A_{1}\cos(mx)\cos(ky)\right] \\ u_{z}=\nu\frac{l_{s}\mu\sin(\mu)-\cos(\mu)}{l_{s}\nu\sinh(\nu)+\cosh(\nu)}\left[-\frac{\sinh(\nu)}{\sin(\mu)}\sin(\mu z)+\sinh(\nu z)\right] \\ \cdot\left[A_{3}\sin(mx)\sin(ky)+A_{4}\sin(mx)\cos(ky)-A_{1}\cos(mx)\sin(ky)-A_{2}\cos(mx)\cos(ky)\right] \end{matrix}$ (II.2)

$\mu=\sqrt{\lambda\mathrm{Re}-m^{2}-k^{2}}, \nu=\sqrt{m^{2}+k^{2}}$

$$\nu\left[l_{s}\nu+\frac{1}{\tanh(\nu)}\right]+\mu\left[l_{s}\mu-\frac{1}{\tan(\mu)}\right]=0$$

Same as the antisymmetric case, $m,k$ cannot both be 0 at the same time.

The 1D HMs may be simply expressed as:

(II.3)

(II.4)

Note that for both the 1D and 2D cases, due to the symmetry property between the *x* and *y* directions, the HMs have two branches, i.e., $x$ branch or $y$ branch where $u_{x}\neq0, u_{y}=0$, or $u_{x}=0$, $u_{y}\neq0$, respectively.

In order to simplify the process of NS equation reduction with the HMs basis (see Part D), below we write down a unified expression for all the 1D, 2D and 3D HMs.

$\begin{matrix} u_{x}=\left(m+\delta_{d,0}\right)\left\{\begin{matrix} \delta_{s,0}\left[\left(\delta_{\kappa,0}\delta_{d,0}+\delta_{d,1}\right)\sin(\mu z)-\delta_{d,1}\frac{l_{s}\mu\cos(\mu)+\sin(\mu)}{l_{s}\nu\cosh(\nu)+\sinh(\nu)}\sinh(\nu z)\right] \\ +\delta_{s,1}\left[\left(\delta_{\kappa,0}\delta_{d,0}+\delta_{d,1}\right)\cos(\mu z)+\delta_{d,1}\frac{l_{s}\mu\sin(\mu)-\cos(\mu)}{l_{s}\nu\sinh(\nu)+\cosh(\nu)}\cosh(\nu z)\right] \end{matrix}\right\} \\ \cdot\left[\delta_{d,1}\left(\delta_{o_{x},0}\sin\left(mx\right)+\delta_{o_{x},1}\cos\left(mx\right)\right)\left(\delta_{o_{y},0}\sin\left(ky\right)+\delta_{o_{y},1}\cos\left(ky\right)\right)+\delta_{d,0}\right] \\ u_{y}=\left(k+\delta_{d,0}\right)\left\{\begin{matrix} \delta_{s,0}\left[\left(\delta_{\kappa,1}\delta_{d,0}+\delta_{d,1}\right)\sin(\mu z)-\delta_{d,1}\frac{l_{s}\mu\cos(\mu)+\sin(\mu)}{l_{s}\nu\cosh(\nu)+\sinh(\nu)}\sinh(\nu z)\right] \\ +\delta_{s,1}\left[\left(\delta_{\kappa,1}\delta_{d,0}+\delta_{d,1}\right)\cos(\mu z)+\delta_{d,1}\frac{l_{s}\mu\sin(\mu)-\cos(\mu)}{l_{s}\nu\sinh(\nu)+\cosh(\nu)}\cosh(\nu z)\right] \end{matrix}\right\} \\ \cdot\left[\delta_{d,1}\left(\delta_{o_{x},1}\sin\left(mx\right)-\delta_{o_{x},0}\cos\left(mx\right)\right)\left(-\delta_{o_{y},1}\sin\left(ky\right)+\delta_{o_{y},0}\cos\left(ky\right)\right)+\delta_{d,0}\right] \\ u_{z}=\nu\left\{\begin{matrix} \delta_{s,0}\frac{l_{s}\mu\cos(\mu)+\sin(\mu)}{l_{s}\nu\cosh(\nu)+\sinh(\nu)}\left[\frac{\cosh(\nu)}{\cos(\mu)}\cos(\mu z)-\cosh(\nu z)\right] \\ +\delta_{s,1}\frac{l_{s}\mu\sin(\mu)-\cos(\mu)}{l_{s}\nu\sinh(\nu)+\cosh(\nu)}\left[-\frac{\sinh(\nu)}{\sin(\mu)}\sin(\mu z)+\sinh(\nu z)\right] \end{matrix}\right\} \\ \cdot\delta_{d,1}\left(\delta_{o_{x},1}\sin\left(mx\right)-\delta_{o_{x},0}\cos\left(mx\right)\right)\left(\delta_{o_{y},0}\sin\left(ky\right)+\delta_{o_{y},1}\cos\left(ky\right)\right) \end{matrix}$(II.5)

Here $\delta$ is the *Kronecker delta* function, and $s,\kappa,d,o_{x},o_{y}$ are the 5 hyper-indices, used to denote different branch of the HMs expression. Their definitions are listed here: $s,\kappa,d,o_{x, y},\in\{0, 1\}$, where $s=0$ denotes the antisymmetric HMs and $s=1$ denotes the symmetric HMs, $\kappa=0$ denotes the $x$ branch and $\kappa=1$ the $y$ branch, $d$ $=0$ denotes the 1D HMs and $d=1$ denotes the 2D or 3D HMs depending on the values of the tuple $\left( m,k \right)$, $o_{x,y}=0$ denotes the $sin(mx,ky)$ branch of $u_{x,y}$ component and $o_{x,y}=1$ denotes the $cos(mx,ky)$ branch of the $u_{x,y}$ branch.

**III. Solving for the eigen-wavevector** $\mu$

We choose the *bisection method* to solve for the eigenvectors $\mu$ with high precision. In order not to miss any of the solutions, it is important to know a-priori the domains in which the solutions lie. In what follows we show that by utilizing the periodic nature of the functions, this is indeed possible.

**2D and 3D Antisymmetric Case**

$\nu\left[l_{s}\nu+\tanh(\nu)\right]+\mu\left[l_{s}\mu+\tan(\mu)\right]=0$ (III.1)

Since we know that $\mu>0$, the left hand side of (51) can be re-written as

$\nu\left[l_{s}\nu+\tanh(\nu)\right]+\mu\left[l_{s}\mu+\tan(\mu)\right]=l_{s}\mu^{2}+\mu\tan\left(\mu\right)+\nu\left[l_{s}\nu+\tanh(\nu)\right]$ (III.2)

For a *given* $\nu$, solving (51) is equivalent to solving the nonlinear equation

$D_{anti}\left(\mu\right)=l_{s}\mu^{2}+\mu\tan\left(\mu\right)+\nu\left[l_{s}\nu+\tanh(\nu)\right]=0$.

Proposition: $D_{anti}\left( \mu\right)=0$ has exactly one zero point in each periodicity $\left[ \left( n-1 \right)\pi, n\pi\right], n\in\mathbf{N}^{+}$, and each zero point is located in the domain $[\left( 2n-1 \right)\pi/2, n\pi]$.

Let us first examine the closed domain $[\left( 2n-1 \right)\pi/2, n\pi]$

$\begin{matrix} \lim_{\mu\to\left(\frac{2n-1}{2}\pi\right)^{+}}D_{anti}\left(\mu\right)=l_{s}\left(\frac{2n-1}{2}\right)^{2}\pi^{2}+\lim_{\mu\to\left(\frac{2n-1}{2}\pi\right)^{+}}\mu\tan\left(\mu\right)+\nu\left[l_{s}\nu+\tanh(\nu)\right]=-\infty\\ D_{anti}\left(n\pi\right)=l_{s}n^{2}\pi^{2}+n\pi\tan\left(n\pi\right)+\nu\left[l_{s}\nu+\tanh(\nu)\right]=l_{s}n^{2}\pi^{2}+\nu\left[l_{s}\nu+\tanh(\nu)\right]>0 \end{matrix}$(III.3)

Since $D_{anti}\left( \mu\right)$ is a continuous function, from the intermediate value theorem we know that there must exist at least one zero point in the domain $[\left( 2n-1 \right)\pi/2, n\pi]$. Meanwhile,

$\begin{matrix} D'_{anti}\left(\mu\right)=2l_{s}\mu+\frac{\mu}{\cos^{2}\left(\mu\right)}+\tan\left(\mu\right)=2l_{s}\mu+\frac{1}{\cos^{2}\left(\mu\right)}\left[\mu+\tan\left(\mu\right)\cos^{2}\left(\mu\right)\right] \\ =2l_{s}\mu+\frac{1}{\cos^{2}\left(\mu\right)}\left[\mu+\frac{1}{2}\sin\left(2\mu\right)\right] \end{matrix}$ . (III.4)

For each $n\in\mathbf{N}^{+}$, it is clear that $\mu\in\left[ \frac{\left( 2n-1 \right)\pi}{2}, n\pi\right]\geq\frac{\pi}{2}>\frac{1}{2}$. Therefore $D_{anti}^{'}\left( \mu\right)>0$ in the domain $[\left( 2n-1 \right)\pi/2, n\pi]$. Hence there exists one and only one zero point in each domain $[\left( 2n-1 \right)\pi/2, n\pi]$, where $n\in\mathbf{N}^{+}$.

Now we look at the domain $[\left( n-1 \right)\pi, \left( 2n-1 \right)\pi/2]$.

$\begin{matrix} \lim_{\mu\to\left(\frac{2n-1}{2}\pi\right)^{-}}D_{anti}\left(\mu\right)=l_{s}\left(\frac{2n-1}{2}\right)^{2}\pi^{2}+\lim_{\mu\to\left(\frac{2n-1}{2}\pi\right)^{-}}\mu\tan\left(\mu\right)+\nu\left[l_{s}\nu+\tanh(\nu)\right]=+\infty\\ D_{anti}\left[\left(n-1\right)\pi\right]=l_{s}\left(n-1\right)^{2}\pi^{2}+\left(n-1\right)\pi\tan\left[\left(n-1\right)\pi\right]+\nu\left[l_{s}\nu+\tanh(\nu)\right] \\ =l_{s}\left(n-1\right)^{2}\pi^{2}+\nu\left[l_{s}\nu+\tanh(\nu)\right]>0 \end{matrix}$ (III.5)

In this domain, $D_{anti}^{'}\left( \mu\right)>0$. Therefore, there can be no zero points. We therefore conclude that the equation $D_{anti}\left( \mu\right)=0$ has exactly one zero point in each periodicity $[\left( n-1 \right)\pi, n\pi]$.

Following the similar procedures, we can solve the eigen-wavevectors of 2D and 3D Symmetric HMs. Here the dispersion relation is given by

$\nu\left[l_{s}\nu+\frac{1}{\tanh(\nu)}\right]+\mu\left[l_{s}\mu-\frac{1}{\tan(\mu)}\right]=0$ (III.6)

For a *given* $\nu$, it is equivalent to solve

$D_{sym}\left(\mu\right)=\nu\left[l_{s}\nu+\frac{1}{\tanh(\nu)}\right]+\mu\left[l_{s}\mu-\frac{1}{\tan(\mu)}\right]=l_{s}\mu^{2}-\mu\cot\left(\mu\right)+\nu\left[l_{s}\nu+\frac{1}{\tanh(\nu)}\right]=0$ (III.7)

Here $\mu=\left( 2n-1 \right)\pi/2, n\in\mathbf{N}^{+}$ are removable singularity points for $1/tan(\mu)$.

Proposition: $D_{sym}\left( \mu\right)=0$ has exactly one zero point in each periodicity $\left[ \left( n-1 \right)\pi, n\pi\right], n\in\mathbf{N}^{+}, n\geq2$, and each zero point is located in the domain $[\left( n-1 \right)\pi, \left( 2n-1 \right)\pi/2]$.

We first examine the domain $[\left( n-1 \right)\pi, \left( 2n-1 \right)\pi/2]$. When $n\geq2$,

$\begin{matrix} \lim_{\mu\to\left[(n-1)\pi\right]^{+}}D_{sym}\left(\mu\right)=l_{s}\left(n-1\right)^{2}\pi^{2}-\lim_{\mu\to\left[(n-1)\pi\right]^{+}}\mu\cot\left(\mu\right)+\nu\left[l_{s}\nu+\frac{1}{\tanh(\nu)}\right]=-\infty\\ D_{sym}\left(\frac{2n-1}{2}\pi\right)=l_{s}\left(\frac{2n-1}{2}\right)^{2}\pi^{2}+\nu\left[l_{s}\nu+\frac{1}{\tanh(\nu)}\right]>0 \end{matrix}$ (III.8)

Since $D_{sym}\left( \mu\right)$ is a continuous function, from the intermediate value theorem we know that there exists at least one zero point in the domain $[\left( n-1 \right)\pi, \left( 2n-1 \right)\pi/2]$. Meanwhile,

$\begin{matrix} D'_{sym}\left(\mu\right)=2l_{s}\mu+\frac{\mu}{\sin^{2}\left(\mu\right)}-\cot\left(\mu\right)=2l_{s}\mu+\frac{1}{\sin^{2}\left(\mu\right)}\left[\mu-\cot\left(\mu\right)\sin^{2}\left(\mu\right)\right] \\ =2l_{s}\mu+\frac{1}{\sin^{2}\left(\mu\right)}\left[\mu-\frac{1}{2}\sin\left(2\mu\right)\right] \end{matrix}$ (III.9)

For each $n\in\mathbf{N}^{+} and n\geq2$, it is clear that the solution $\mu\in[\left( n-1 \right)\pi, \left( 2n-1 \right)\pi/2]\geq\pi>\frac{1}{2}$. If $n=1$, for $\mu\in\left[ 0,\frac{\pi}{2} \right]$,

$$\frac{d}{d\mu}\left[\mu-\frac{1}{2}\sin\left(2\mu\right)\right]=1-\cos\left(2\mu\right)\geq0, \to\mu-\frac{1}{2}\sin\left(2\mu\right)\geq0$$

Therefore $D_{anti}^{'}\left( \mu\right)>0$ in each domain $\left[ \left( n-1 \right)\pi, \frac{\left( 2n-1 \right)\pi}{2} \right], n\in\mathbf{N}^{+}$. It follows that there exists one and only one zero point in each domain $\left[ \left( n-1 \right)\pi, \frac{\left( 2n-1 \right)\pi}{2} \right]$ , where $n\in\mathbf{N}^{+}$.

Now let us examine the closed domain $[\left( 2n-1 \right)\pi/2, n\pi], n\in\mathbf{N}^{+}$.

$\begin{matrix} \lim_{\mu\to n\pi^{-}}D_{sym}\left(\mu\right)=l_{s}n^{2}\pi^{2}-\lim_{\mu\to\left(n\pi\right)^{-}}\mu\cot\left(\mu\right)+\nu\left[l_{s}\nu+\tanh(\nu)\right]=+\infty\\ D_{sym}\left(\frac{2n-1}{2}\pi\right)=l_{s}\left(\frac{2n-1}{2}\right)^{2}\pi^{2}+\nu\left[l_{s}\nu+\frac{1}{\tanh(\nu)}\right]>0 \end{matrix}$ (III.10)

In this domain, $D_{sym}^{'}\left( \mu\right)>0$. Therefore, there exist no zero points.

For the domain $\left[ 0,\pi\right]$,

$\begin{matrix} \lim_{\mu\to0^{+}}D_{sym}\left(\mu\right)=-\lim_{\mu\to0^{+}}\mu\cot\left(\mu\right)+\nu\left[l_{s}\nu+\frac{1}{\tanh(\nu)}\right]\geq-1+\frac{\nu}{\tanh(\nu)}\geq-1+\nu\\ \lim_{\mu\to\pi^{-}}D_{sym}\left(\mu\right)=l_{s}\pi^{2}-\lim_{\mu\to\left(\pi\right)^{-}}\mu\cot\left(\mu\right)+\nu\left[l_{s}\nu+\tanh(\nu)\right]=+\infty\end{matrix}$ (III.11)

Therefore as along as $\nu>1, i.e., m>1 \mathrm{or} k>1$, there exists no solution in the first period.

Hence $D_{sym}\left( \mu\right)=0$ has exactly one zero point in each period $[\left( n-1 \right)\pi, n\pi] n\in\mathbf{N}^{+}$.

**1D Antisymmetric HMs**

$D\left(\mu\right)=l_{s}\mu+\tan(\mu)=0, l_{s}\ll1$ (III.12)

We assume that the slip length is much smaller than 1, and $D\left( \mu\right)$ is a continuous function so that

$\begin{matrix} D\left[\left(n-1\right)\pi\right]=l_{s}\left(n-1\right)\pi+\tan\left[\left(n-1\right)\pi\right]=l_{s}\left(n-1\right)\pi\geq0 \\ \lim_{\mu\to\left(\frac{2n-1}{2}\pi\right)^{-}}D\left(\mu\right)=l_{s}\left(\frac{2n-1}{2}\pi\right)+\lim_{\mu\to\left(\frac{2n-1}{2}\pi\right)^{-}}\tan\left(\mu\right)=+\infty\\ \lim_{\mu\to\left(\frac{2n-1}{2}\pi\right)^{+}}D\left(\mu\right)=l_{s}\left(\frac{2n-1}{2}\pi\right)+\lim_{\mu\to\left(\frac{2n-1}{2}\pi\right)^{+}}\tan\left(\mu\right)=-\infty\\ D\left(n\pi\right)=l_{s}n\pi+\tan\left(n\pi\right)=l_{s}n\pi\geq0 \end{matrix}$ (III.13)

the derivative of $D(\mu)$ is positive definite

$D'\left(\mu\right)=l_{s}+\frac{1}{\cos^{2}\left(\mu\right)}\geq0$.

Therefore there exists only one zero point in each of the domains $\left[ \frac{\left( 2n-1 \right)\pi}{2}, n\pi\right], n\in\mathbf{N}^{+}$.

**1D Symmetric HMs**

Let

$D\left(\mu\right)=\cot(\mu)-l_{s}\mu=0$ (III.14)

Since $D\left( \mu\right)$ is a continuous function, and

$\begin{matrix} \lim_{\mu\to\left[\left(n-1\right)\pi\right]^{+}}D\left(\mu\right)=-l_{s}\left(n-1\right)\pi+\lim_{\mu\to\left[\left(n-1\right)\pi\right]^{+}}\cot\left(\mu\right)=+\infty\\ D\left(\frac{2n-1}{2}\pi\right)=-l_{s}\left(\frac{2n-1}{2}\pi\right)+\cot\left(\frac{2n-1}{2}\pi\right)=-l_{s}\left(\frac{2n-1}{2}\pi\right)\leq0 \\ \lim_{\mu\to n\pi^{-}}D\left(\mu\right)=-l_{s}n\pi+\lim_{\mu\to n\pi^{-}}\cot\left(\mu\right)=-\infty\end{matrix}$ (III.15)

The derivative of $D(\mu)$ is given by $D^{'}\left( \mu\right)=-\frac{1}{\sin^{2} \left( \mu\right)}-l_{s}\leq0$, therefore there exists one and only one zero point in each of the domains $\left[ \left( n-1 \right)\pi, \frac{\left( 2n-1 \right)\pi}{2} \right], n\in\mathbf{N}^{+}$.

## IV. Reduction of the Navier-Stokes equation by using the hydrodynamic modes

The perturbed incompressible Navier Stokes equation of Poiseuille flow takes the form as

$\begin{matrix} \frac{\partial u}{\partial t}+\left(u+u^{P}\left(\mathbf{r}\right)\right)\cdot\nabla\left(u+u^{P}\left(\mathbf{r}\right)\right)=-\nabla\left(p_{0}+p\right)+\frac{1}{\mathrm{Re}}\nabla^{2}\left(u+u^{P}\left(\mathbf{r}\right)\right) \\ \nabla\cdot\left(u+u^{P}\left(\mathbf{r}\right)\right)=0 \end{matrix}$ (IV.1)

where $u^{P}\left(\mathbf{r}\right)\text{=}\left(1-z^{2}+2l_{s},0,0\right)$ is the steady state solution of Poiseuille flow, with the corresponding pressure field given by

$\nabla p_{0}=\left(-P_{0},0,0\right)=\left(-\frac{2}{\mathrm{Re}},0,0\right).$ (IV.2)

The full nonlinear perturbation equation takes the form as

$\frac{\partial u}{\partial t}=-\left(u^{P}\left(\mathbf{r}\right)\cdot\nabla\right)u-\left(u\cdot\nabla\right)u^{P}\left(\mathbf{r}\right)-\nabla p+\frac{1}{\mathrm{Re}}\nabla^{2}u-\left(u\cdot\nabla\right)u$ . (IV.3)

Here $u\left(r,t\right)$ is the perturbed velocity vector field at time $t$. Given the initial perturbation $u\left(r,0\right)$at *t*=0, the velocity field will evolve with time $t$. Due to the completeness and mutual orthogonality of the HMs, we expand the velocity field in terms of the HMs as

$u\left(r,t\right)=\sum_{\alpha=1}^{\infty} c_{\alpha}\left(t\right)u_{\alpha}\left(\mathbf{r}\right), c_{\alpha}\left(t\right), u_{\alpha}\left(\mathbf{r}\right)\text{ }\in\mathbf{R}^{3}$ (IV.4)

Through projection, we obtain the coupled autonomous ODEs for the expansion coefficients

(IV.5)

We assume that all perturbation variables $p\left(r,t\right),u\left(r,t\right)$ are periodic in the *xy* plane. Under this assumption

$\left\langle u_{\gamma}\left(\mathbf{r}\right),\nabla p\right\rangle=\left\langle u_{\gamma}\left(\mathbf{r}\right),\nabla p\right\rangle+\left\langle\nabla\cdot u_{\gamma}\left(\mathbf{r}\right),p\right\rangle=\int\nabla\cdot\left(pu_{\gamma}\left(\mathbf{r}\right)\right)d\Omega=\int pu_{\gamma}\left(\mathbf{r}\right)\cdot\partial\mathbf{\Omega}\equiv0.$ (IV.6)

Here $d\Omega$ denotes volume integral, and $\partial\mathbf{\Omega}$denotes the integration over the surface of the relevant volume, with the unit outward vector normal to the surface. The surface integral is zero because the normal velocity is zero at the upper and lower boundary, and incompressibility condition guarantees that the in-flow must be equal to the out-flow along the *yz* plane or the *xz* plane. Hence the pressure $p$ is eliminated from the NS equation to obtain the following coupled matrix nonlinear ODEs:

$\begin{matrix} \frac{\partial c_{\gamma}\left(t\right)}{\partial t}=-\sum_{\alpha=1}^{\infty} \left\langle u_{\gamma}\left(\mathbf{r}\right),\left(u^{P}\left(\mathbf{r}\right)\cdot\nabla\right)u_{\alpha}\left(\mathbf{r}\right)\right\rangle c_{\alpha}\left(t\right)-\sum_{\alpha=1}^{\infty} \left\langle u_{\gamma}\left(\mathbf{r}\right),\left(u_{\alpha}\left(\mathbf{r}\right)\cdot\nabla\right)u^{P}\left(\mathbf{r}\right)\right\rangle c_{\alpha}\left(t\right) \\ +\frac{1}{\mathrm{Re}}\sum_{\alpha=1}^{\infty} \left\langle u_{\gamma}\left(\mathbf{r}\right),\nabla^{2}u_{\alpha}\left(\mathbf{r}\right)\right\rangle c_{\alpha}\left(t\right)-\sum_{\alpha=1}^{\infty} \sum_{\beta=1}^{\infty} \left\langle u_{\gamma}\left(\mathbf{r}\right),\left(u_{\alpha}\left(\mathbf{r}\right)\cdot\nabla\right)u_{\beta}\left(\mathbf{r}\right)\right\rangle c_{\alpha}\left(t\right)c_{\beta}\left(t\right) \end{matrix}$ . (IV.7)

The above equation set can be written into the matrix form as

, (IV.8)

where represents the coefficients matrix of the linear terms, and represents a 3^rd^ order tensor. In component form, this autonomous equation can be expressed as

(IV.9)

The elements of the two operators can be evaluated as follows:

(IV.10a)

(IV.10b)

Since $u_{\alpha}$has the simple analytic forms, the evaluation of the above integrals can be carried out in an automated manner by using suitable softwares. The resulting elements are also analytic in nature.

In the range of small Re, the real parts of the eigenvalues of the linear operatorare always negative. However, as Re increases, one of the eigenvalues’ real part can cross zero at a critical set of values {Re_c_, *m*_c_} that signals instability of the channel flow. At this critical point, the accompanying eigenstate can be easily obtained as a set of$\{u_{\alpha}\}_{c}$. Below we show a video illustration of this oscillating critical state, which comprises a large vortex with an anti-vortex oscillating at a fixed frequency. Each vortex has a lateral dimension given by . The oscillation frequency is given by the imaginary part of the complex critical eigenvalue $\sigma_{c}$, Imag ($\sigma_{c}$) $\approx$ 0.269.

## V. Explicit 4^th^ order Runge-Kutta scheme for the time evolution of the autonomous ODEs

Let $\mathbf{g}$ be a vector of size $N$ with its elements $\text{g}_{\gamma}:=\mathbf{c}^{T}\cdot N_{ , ,\gamma}\cdot\mathbf{c}$. The time evolution ODEs of the HMs projection coefficients vector $\mathbf{c}$ takes the form

and the generalized formula of the explicit Runge-Kutta scheme is

$k_{1}=f\left[\mathbf{c}^{n}\right], k_{2}=f\left[\mathbf{c}^{n}+\Delta ta_{21}k_{1}\right], ... , k_{s}=f\left[\mathbf{c}^{n}+\Delta t\sum_{i=1}^{s-1} a_{si}k_{i}\right], \mathbf{c}^{n+1}=\mathbf{c}^{n}+\Delta t\sum_{i=1}^{s} b_{i}k_{i}$ (V.2)

where $\left\{a_{ij}\right\}_{i,j=1,2,...,s}$ denotes the Runge-Kutta (RK) coefficients matrix, $\left\{k_{i}\right\}_{i=1,2,...,s}$ denotes the intermediate vectors, and $b_{i}'s$are the coefficients needed to fulfill the RK scheme. We denote $c_{k}^{n}$ as the *k*^th^ component of vector $\mathbf{c}$ at time step $n$ and choose the even discrete time step size to be $\Delta t$. Here *s* denotes the order of the scheme.

For $s=4$, we illustrate how to implement the explicit time evolutions for the coefficients vector $\mathbf{c}$ with the precision preserved to the order of $\left( \Delta t \right)^{4}$. Our numerical expriments are based on this set 4^th^ order explicit scheme. The main work is to obtain the values of the Runge-Kutta coefficients matrix as well as the corresponding vector series $\left\{k_{i}\right\}_{i=1,2,3,4}$.

Substituting $s=4$ into (V.2) yields

$\begin{matrix} \mathbf{c}^{n+1}=\mathbf{c}^{n}+\Delta t\sum_{i}^{4} b_{i}\mathbf{k}_{i} \\ \mathbf{k}_{1}=\mathbf{f}\left[\mathbf{c}^{n}\right], \mathbf{k}_{2}=\mathbf{f}\left[\mathbf{c}^{n}+\Delta ta_{21}\mathbf{k}_{1}\right], \mathbf{k}_{3}=\mathbf{f}\left[\mathbf{c}^{n}+\Delta ta_{31}\mathbf{k}_{1}+\Delta ta_{32}\mathbf{k}_{2}\right], \mathbf{k}_{4}=\mathbf{f}\left[\mathbf{c}^{n}+\Delta ta_{41}\mathbf{k}_{1}+\Delta ta_{42}\mathbf{k}_{2}+\Delta ta_{43}\mathbf{k}_{3}\right], \end{matrix}$(V.3)

Further simplification can be made by setting$a_{31}=a_{41}=a_{42}=0$ to eliminate the redundant degrees of freedom:

$\mathbf{k}_{1}=\mathbf{f}\left[\mathbf{c}^{n}\right], \mathbf{k}_{2}=\mathbf{f}\left[\mathbf{c}^{n}+\Delta ta_{21}\mathbf{k}_{1}\right], \mathbf{k}_{3}=\mathbf{f}\left[\mathbf{c}^{n}+\Delta ta_{32}\mathbf{k}_{2}\right], \mathbf{k}_{4}=\mathbf{f}\left[\mathbf{c}^{n}+\Delta ta_{43}\mathbf{k}_{3}\right], \mathbf{c}^{n+1}=\mathbf{c}^{n}+\Delta t\sum_{i}^{4} b_{i}\mathbf{k}_{i}$ (V.4)

By Taylor expansion,

(V.5)

(V.4) can be written as

$c_{k}^{n+1}=c_{k}^{n}+\Delta tb_{1}f_{k}\left(\mathbf{c}^{n}\right)+\Delta tb_{2}f_{k}\left(\mathbf{c}^{n}+\Delta ta_{21}\mathbf{k}_{1}\right)+\Delta tb_{3}f_{k}\left(\mathbf{c}^{n}+\Delta ta_{32}\mathbf{k}_{2}\right)+\Delta tb_{4}f_{k}\left(\mathbf{c}^{n}+\Delta ta_{43}\mathbf{k}_{3}\right)$(V.6a)

By using (V.4), the vectors $\mathbf{k}_{\mathbf{1}}\boldsymbol{,}\mathbf{k}_{\mathbf{2}}\boldsymbol{,}\mathbf{k}_{\mathbf{3}}$ can be further expanded as follows:

$\begin{matrix} \left(\mathbf{k}_{2}\right)_{k}=f_{k}\left(\mathbf{c}^{n}+\Delta ta_{21}\mathbf{k}_{1}\right) \\ =f_{k}\left(\mathbf{c}^{n}\right)+\Delta ta_{21}\sum_{m}^{N} \frac{\partial f_{k}}{\partial c_{m}}f_{m}+\frac{1}{2}\left(\Delta ta_{21}\right)^{2}\sum_{m}^{N} \sum_{n}^{N} \frac{\partial^{2}f_{k}}{\partial c_{m}\partial c_{n}}f_{m}f_{n} \\ +\left(\Delta ta_{21}\right)^{3}\sum_{\alpha_{1}+\alpha_{2}+...+\alpha_{N}=3} \sum_{i}^{s} \frac{1}{\alpha_{1}!...\alpha_{N}!}\frac{\partial^{3}f_{k}}{\partial c_{1}^{\alpha_{1}}...\partial c_{N}^{\alpha_{N}}}\Pi_{m}^{N}\left(\mathbf{k}_{1}\right)_{m}^{\alpha_{m}}... \end{matrix}$ (V.6b)

$\begin{matrix} \left(\mathbf{k}_{3}\right)_{k}=f_{k}\left(\mathbf{c}^{n}+\Delta ta_{32}\mathbf{k}_{2}\right) \\ =f_{k}\left(\mathbf{c}^{n}\right)+\Delta ta_{32}\sum_{m}^{N} \frac{\partial f_{k}}{\partial c_{m}}\left(\mathbf{k}_{2}\right)_{m}+\frac{1}{2}\left(\Delta ta_{32}\right)^{2}\sum_{m}^{N} \sum_{n}^{N} \frac{\partial^{2}f_{k}}{\partial c_{m}\partial c_{n}}\left(\mathbf{k}_{2}\right)_{m}\left(\mathbf{k}_{2}\right)_{n} \\ +\left(\Delta ta_{32}\right)^{3}\sum_{\alpha_{1}+\alpha_{2}+...+\alpha_{N}=3} \sum_{i}^{s} \frac{1}{\alpha_{1}!...\alpha_{N}!}\frac{\partial^{3}f_{k}}{\partial c_{1}^{\alpha_{1}}...\partial c_{N}^{\alpha_{N}}}\Pi_{m}^{N}\left(\mathbf{k}_{2}\right)_{m}^{\alpha_{m}}... \\ \end{matrix}$ (V.6c)

$\begin{matrix} \left(\mathbf{k}_{4}\right)_{k}=f_{k}\left(\mathbf{c}^{n}+\Delta ta_{43}\mathbf{k}_{3}\right) \\ =f_{k}\left(\mathbf{c}^{n}\right)+\Delta ta_{43}\sum_{m}^{N} \frac{\partial f_{k}}{\partial c_{m}}\left(\mathbf{k}_{3}\right)_{m}+\frac{1}{2}\left(\Delta ta_{43}\right)^{2}\sum_{m}^{N} \sum_{n}^{N} \frac{\partial^{2}f_{k}}{\partial c_{m}\partial c_{n}}\left(\mathbf{k}_{3}\right)_{m}\left(\mathbf{k}_{3}\right)_{n} \\ +\left(\Delta ta_{43}\right)^{3}\sum_{\alpha_{1}+\alpha_{2}+...+\alpha_{N}=3} \sum_{i}^{s} \frac{1}{\alpha_{1}!...\alpha_{N}!}\frac{\partial^{3}f_{k}}{\partial c_{1}^{\alpha_{1}}...\partial c_{N}^{\alpha_{N}}}\Pi_{m}^{N}\left(\mathbf{k}_{3}\right)_{m}^{\alpha_{m}}... \end{matrix}$ (V.6d)

We denote the *m*^th^ component of vector $\mathbf{k}_{l}$ as $\left(\mathbf{k}_{l}\right)_{m}$, with $\alpha_{n}$ denoting the Taylor expansion power exponents of the *n*^th^ coordinate component $c_{n}$.

By substituting (V.6b-d) into (V.6a), we obtain (V.7)

By grouping the terms according to their $\Delta t$ orders and comparing the coefficients in (V.7) of the same order with those in (V.5), we obtain

It is easy to check that one of the possible parameters set is given by

$b_{1}=\frac{1}{6},b_{2}=\frac{1}{3}, b_{3}=\frac{1}{3}, b_{4}=\frac{1}{6}, a_{21}=\frac{1}{2}, a_{32}=\frac{1}{2}, a_{43}=1$.

VI. Energy conservation equation expressed in terms of the hydrodynamic modes

In this part, we derive the energy conservation law expressed in terms of the HMs. We denote the initial perturbation to be $u\left(r,0\right)$ and the unperturbed steady Poiseuille flow to be $u^{P}\left(r\right)$. The time evolution of the full NS equation has the form

$\frac{\partial\left(u^{P}\left(r\right)+u\right)}{\partial t}+\left[\left(u^{P}\left(r\right)+u\right)\cdot\nabla\right]\left(u^{P}\left(r\right)+u\right)=-\nabla\left(P_{0}+p\right)+\frac{1}{\mathrm{Re}}\nabla^{2}\left(u^{P}\left(r\right)+u\right),$ (VI.1)

where $u^{P}\left(r\right)\text{ is along the }x\text{ direction only}$. We apply the periodic boundary conditions:

$\begin{matrix} u\left(-L,t\right)=u\left(L,t\right), p\left(-L,t\right)=p\left(L,t\right) \\ u\left(-W,t\right)=u\left(W,t\right), p\left(-W,t\right)=p\left(W,t\right) \end{matrix}$. (VI.2)

Here $L$ is channel’s length along the $x$ axis and $W$ is channel’s width along the $y$ axis. Both are expressed with the length unit of height $H$, taken to be 1 in this work.

It is observed that

$\begin{matrix} u^{P}\left(r\right)=1-z^{2}+2l_{s}, \\ \frac{\partial u^{P}\left(r\right)}{\partial t}=0=-\nabla P_{0}+\frac{1}{\mathrm{Re}}\nabla^{2}u^{P}\left(r\right)=-\nabla P_{0}-\frac{2}{\mathrm{Re}}, \\ \frac{\partial P_{0}}{\partial x}=-\frac{2}{\mathrm{Re}}, \\ \frac{\partial P_{0}}{\partial y}=\frac{\partial P_{0}}{\partial z}=0, \\ \frac{\partial u}{\partial t}=-u^{P}\left(r\right)\frac{\partial u}{\partial x}-\left(u\cdot\nabla\right)u^{P}\left(r\right)-\left(u\cdot\nabla\right)u-\nabla p+\frac{1}{\mathrm{Re}}\nabla^{2}u \end{matrix}$ (VI.3)

The total kinetic energy of the channel flow evolves as a function of time $t$ as

$\begin{matrix} \frac{1}{2}\frac{d}{dt}\int\left|u^{P}\left(r\right)+u\right|^{2}d\Omega=-\left\langle\nabla\left(P_{0}+p\right),\left(u^{P}\left(r\right)+u\right)\right\rangle+\frac{1}{\mathrm{Re}}\left\langle\left(u^{P}\left(r\right)+u\right),\nabla^{2}\left(u^{P}\left(r\right)+u\right)\right\rangle\\ =\frac{2}{\mathrm{Re}}\int\left(u^{P}\left(r\right)+u\right)d\Omega+\frac{1}{\mathrm{Re}}\left\langle\left(u^{P}\left(r\right)+u\right),\nabla^{2}\left(u^{P}\left(r\right)+u\right)\right\rangle\end{matrix}$, (VI.4)

where the nonlinear convection term disappears under the periodic boundary condition as can be shown below:

$$\left\langle\left[\left(u^{P}\left(r\right)+u\right)\cdot\nabla\right]\left(u^{P}\left(r\right)+u\right),u^{P}\left(r\right)+u\right\rangle=\frac{1}{2}\left\langle\nabla\left|u^{P}\left(r\right)+u\right|^{2}-2\left(u^{P}\left(r\right)+u\right)\times\left[\nabla\times\left(u^{P}\left(r\right)+u\right)\right],u^{P}\left(r\right)+u\right\rangle=0$$

This results from the following two identities:

$\left\langle\nabla\left|u^{P}\left(r\right)+u\right|^{2},u^{P}\left(r\right)+u\right\rangle=\int\nabla\cdot\left[\left|u^{P}\left(r\right)+u\right|^{2}\left(u^{P}\left(r\right)+u\right)\right]d\Omega=0$,

and

$-\left\langle\left(u^{P}\left(r\right)+u\right)\times\left[\nabla\times\left(u^{P}\left(r\right)+u\right)\right],\left(u^{P}\left(r\right)+u\right)\right\rangle=0$.

In (VI.4), the first term on the right hand side represents the rate of energy input exerted by the external pressure. We denote it as $W_{p}(t)$. The second term on the right hand side represents the rate of viscous dissipation, and we denote it as $W_{d}\left( t \right)$. Since we know that all HMs have zero net momentum except for the 1D symmetric HMs, thus $W_{p}$ can be further simplified as

$W_{p}\left(t\right)=\frac{2}{\mathrm{Re}}\int\left(u^{P}\left(r\right)+u_{1D,\text{sym}}\right)d\Omega$ . (VI.5)

Energy conservation law states that

$\frac{dE_{kinetic}\left(t\right)}{dt}=W_{p}\left(t\right)-W_{d}\left(t\right)$. (VI.6)

To facilitate the calculation of the energy dissipation rate, we would like to project the steady flow $u^{P}\left(r\right)$ onto the 1D symmetric HMs. The projection coefficient can be simply calculated as:

. (VI.7)

In this way the total velocity field $v\left(r,t\right)=u^{P}\left(r\right)+u\left(r,t\right)$ can be completely expanded in terms of the HMs:

$\begin{matrix} u^{P}\left(r\right)+u\left(r,t\right)=\sum_{\alpha=1}^{N} w_{\alpha}\left(t\right)u_{\alpha}\left(r\right), \\ w_{\alpha}\left(t\right)=g_{\alpha}+c_{\alpha}\left(t\right) , \end{matrix}$ (VI.8)

so that

$W_{d}\left(t\right)=\frac{1}{\mathrm{Re}}\int\left(\sum_{\alpha=1}^{N} w_{\alpha}\left(t\right)u_{\alpha}\left(r\right)\right)\cdot\left(\nabla^{2}\sum_{\alpha=1}^{N} w_{\alpha}\left(t\right)u_{\alpha}\left(r\right)\right)dxdydz$. (VI.9)

But since

$-\nabla p_{\alpha}+\frac{1}{\mathrm{Re}}\nabla^{2}u_{\alpha}\left(r\right)=-\lambda_{\alpha}u_{\alpha}\left(r\right)$,

we have

$-\nabla\sum_{\alpha=1}^{N} w_{\alpha}\left(t\right)p_{\alpha}+\frac{1}{\mathrm{Re}}\nabla^{2}\sum_{\alpha=1}^{N} w_{\alpha}\left(t\right)u_{\alpha}\left(r\right)=-\sum_{\alpha=1}^{N} \lambda_{\alpha}w_{\alpha}\left(t\right)u_{\alpha}\left(r\right)$.

$$\begin{matrix} -\left\langle\nabla\sum_{\alpha=1}^{N} w_{\alpha}\left(t\right)p_{\alpha},\sum_{\alpha=1}^{N} w_{\alpha}\left(t\right)u_{\alpha}\left(r\right)\right\rangle+\frac{1}{\mathrm{Re}}\left\langle\nabla^{2}\sum_{\alpha=1}^{N} w_{\alpha}\left(t\right)u_{\alpha}\left(r\right),\sum_{\alpha=1}^{N} w_{\alpha}\left(t\right)u_{\alpha}\left(r\right)\right\rangle=\frac{1}{\mathrm{Re}}\left\langle\nabla^{2}\sum_{\alpha=1}^{N} w_{\alpha}\left(t\right)u_{\alpha}\left(r\right),\sum_{\alpha=1}^{N} w_{\alpha}\left(t\right)u_{\alpha}\left(r\right)\right\rangle\\ =\left\langle-\sum_{\alpha=1}^{N} \lambda_{\alpha}w_{\alpha}\left(t\right)u_{\alpha}\left(r\right),\sum_{\alpha=1}^{N} w_{\alpha}\left(t\right)u_{\alpha}\left(r\right)\right\rangle\\ =-\sum_{\alpha=1}^{N} \lambda_{\alpha}w_{\alpha}^{2}\left(t\right)\left\|u_{\alpha}\left(r\right)\right\|^{2} \end{matrix}$$

In the above, we have used (IV.6), with the periodic boundary condition, to eliminate the pressure term.

In this manner the energy dissipation rate $W_{d}(t)$ can be expressed as

$W_{d}\left(t\right)=-\sum_{\alpha=1}^{N} w_{\alpha}^{2}\left(t\right)\lambda_{\alpha}\int\left\|u_{\alpha}\left(r\right)\right\|^{2}dxdydz$ , (VI.10a)

and

$W_{p}\left(t\right)=\frac{2}{\mathrm{Re}}\times4LW\times\left(\sum_{\alpha\in\left\{\begin{matrix} \text{1D symmetric} \\ \text{HMs index} \end{matrix}\right\}} w_{\alpha}\left(\tau\right)\int_{-1}^{1} u_{\alpha}\left(r\right)dz\right)$ . (VI.10b)

The energy conservation equation can be expressed in terms of the HMs as

$\begin{matrix} \frac{dE_{kinetic}}{dt}=\frac{2}{\mathrm{Re}}\times4LW\times\left(\sum_{\alpha\in\left\{\begin{matrix} \text{1D symmetric} \\ \text{HMs index} \end{matrix}\right\}} w_{\alpha}\left(t\right)\int_{-1}^{1} u_{\alpha}\left(r\right)dz\right)-\sum_{\alpha=1}^{N} w_{\alpha}^{2}\left(t\right)\lambda_{\alpha}\int\left\|u_{\alpha}\left(r\right)\right\|^{2}dxdydz \\ \end{matrix}$ . (VI.11)

The second term on the right hand side of (VI.11) represents the combined viscous dissipation of the Poiseuille flow and the 1D symmetric HMs. This can be seen from the definition of $w_{\alpha}$, (VI.8). Hence once the 1D symmetric modes appear above the Re_c_, the work done by the externally applied pressure, represented by the first term on the right hand side, will inevitably be shared between the Poiseuille flow and the 1D symmetric modes. Hence the Poiseuille flow rate must decrease as a result.
